# Emetine dihydrochloride inhibits Chikungunya virus nsP2 helicase and shows robust antiviral activity in cells and mice

**DOI:** 10.1186/s12929-026-01280-9

**Published:** 2026-07-20

**Authors:** Anshula Sharma, Chandru Subramani, K. A. Shouri, Ghanshyam Sharma, Brohmomoy Basu, Abhay Deep Pandey, Archana Rout, Devendra Sharma, Deepti Jain, Sudhanshu Vrati

**Affiliations:** 1 Regional Centre for Biotechnology, Faridabad, 121 001 India; 2 Present Address: Department of Pathology, University of Texas Medical Branch, Galveston, TX 77550 USA; 3 Present Address: Indian Institute of Technology Dharwad, Dharwad, 580 007 India; 4 Present Address: Indian Institute of Technology Roorkee, Roorkee, 247 667 India

**Keywords:** Mouse model, RNA replication, Microscale thermophoresis

## Abstract

**Background:**

Chikungunya virus (CHIKV), a mosquito-borne alphavirus, causes frequent global epidemics of chikungunya fever, characterized by severe joint pain and debilitating arthritis. With no specific antiviral therapies available, these outbreaks pose a major public health challenge, particularly in tropical regions. There is thus urgent need to develop novel antivirals. Drug repurposing is an attractive strategy to identify potential antivirals targeting CHIKV replication.

**Methods:**

The Spectrum collection of approved drugs was screened using high-content cell imaging to identify potential CHIKV replication inhibitors. Efficacy of these CHIKV inhibitors was evaluated in a C57BL/6 mouse model of chikungunya disease, monitoring viremia and clinical symptoms like joint swelling. The CHIKV inhibitor's effect on virus uptake, replication, RNA synthesis, and protein production was studied in ERMS cells. In silico molecular docking was used to study the inhibitor’s binding to the CHIKV proteins. Binding was validated in vitro using microscale thermophoresis and isothermal titration calorimetry. CHIKV helicase, protease, and ATPase activities were measured in the presence of emetine dihydrochloride (ED) to determine its mechanism of action.

**Results:**

Out of the four CHIKV inhibitors identified by high content screening, ED potently inhibited CHIKV replication in the mouse model, yielding significantly lower viremia levels. Notably, CHIKV-infected mice treated with ED showed no clinical symptoms of joint swelling. In ERMS cells, ED blocked CHIKV uptake and early replication by suppressing viral RNA synthesis, which in turn prevented viral protein production. Computational modelling predicted ED’s binding around the RNA-binding site of the CHIKV nsP2 helicase domain. In vitro validation confirmed dose-dependent ED binding with CHIKV nsP2. ED specifically inhibited helicase unwinding in a concentration-dependent manner without affecting ATPase or protease activity.

**Conclusions:**

This study demonstrates that ED inhibits CHIKV replication in cultured cells and a mouse model of infection through binding to CHIKV nsP2 and inhibition of its helicase activity. While this is a promising virus-targeted mechanism of ED’s antiviral action, additional host-directed mechanisms warrant further investigation.

**Supplementary Information:**

The online version contains supplementary material available at 10.1186/s12929-026-01280-9.

## Introduction

Chikungunya virus (CHIKV) is an arthropod-borne alphavirus of the *Togaviridae* family, transmitted primarily by *Aedes aegypti* and *Aedes albopictus* mosquitoes. First identified during a 1952–1953 outbreak in Tanzania [1], CHIKV has since caused widespread epidemics across Africa, Asia, the Americas, Europe, and Australia [2]. Major outbreaks include recurrent epidemics in Africa and Asia (1960s–2000s), the 2005–2006 Indian Ocean epidemic (notably on Réunion Island), and a large outbreak in India in 2006 affecting ~ 1.25 million people. The virus spread to the Western Hemisphere during 2013–2014, causing extensive outbreaks in the Caribbean and South America. CHIKV has now been circulating globally, reaching over 110 countries. In 2024, approximately 480,000 cases and more than 200 deaths were reported worldwide as of November 30 [3]. In addition to urban transmission in humans, CHIKV is maintained in a sylvatic cycle involving non-human primates, which act as reservoirs during inter-epidemic periods [1].

CHIKV infection in humans typically presents as an acute febrile illness characterized by high fever, maculopapular rash, myalgia, polyarthralgia, and headache. While most symptoms resolve within a week, joint pain can persist for months or even years in some patients [4, 5]. Although the disease is generally self-limiting with a low fatality rate (~ 0.1%), chronic joint complications frequently lead to long-term disability, significantly impacting quality of life and imposing substantial socioeconomic burdens, particularly in low- and middle-income countries [6]. Additionally, the potential for vertical transmission, viral evolution, increased human mobility, and geographic expansion highlight the ongoing public health threat posed by CHIKV and underscores the need for effective antiviral interventions [7].

CHIKV possesses a ~ 11.8 kb positive-sense single-stranded RNA genome with a 5′ cap and a 3′ poly(A) tail [8]. The genome encodes two polyproteins: a non-structural polyprotein, which is cleaved into nsP1–nsP4, and a structural polyprotein, processed into capsid, E3, E2, 6 K, and E1 proteins. The non-structural proteins drive viral replication: nsP1 mediates RNA capping, nsP2 functions as a protease and helicase, nsP3 supports replication complex formation, and nsP4 acts as the RNA-dependent RNA polymerase. Structural proteins are essential for virion assembly and entry, with capsid packaging the genome, E2 mediating receptor binding, E1 enabling membrane fusion, E3 assisting glycoprotein maturation, and 6 K facilitating protein trafficking and assembly [9, 10]. Together, these proteins coordinate CHIKV replication, assembly, and release.

Several viral proteins have been investigated as potential targets for direct-acting antiviral development [11–16]. Various host-targeting antivirals have also been identified by targeting specific host proteins directly involved in the virus replication process [17–21]. High-throughput cell-based screening of chemical and natural compound libraries, as well as computational and virtual screening strategies, have identified numerous candidate inhibitors [15, 16, 22–28]. However, most remain at early stages and require validation in animal models and clinical trials. Despite these efforts, no approved antiviral therapies exist, highlighting the need for continued research to develop effective CHIKV treatments.

In this study, a cell-based high-throughput screen of 2,560 compounds from the Spectrum Collection identified four antiviral candidates: niclosamide, gambogic acid, celastrol, and emetine dihydrochloride (ED). Among these, ED showed the highest potency, with low cytotoxicity and strong antiviral activity at nanomolar concentrations in vitro. In a mouse model, ED significantly reduced viremia and CHIKV-induced joint swelling. Mechanistically, ED was found to bind the viral nsP2 protein, inhibiting its RNA helicase activity and thereby suppressing viral replication.

## Material and methods

### Ethics statement

For the animal experiments, the guidelines on the care and use of laboratory animals provided by the Committee for the Purpose of Control and Supervision of Experiments on Animals (CPCSEA), Government of India, were followed. The experimental protocol was approved by the Institutional Animal Ethics Committee of the Regional Centre for Biotechnology (RCB/IAEC/2019/047).

### Cell lines and viruses

BHK-21 (baby hamster kidney cells) and Vero (African green monkey kidney cells) cell lines were obtained from the National Centre for Cell Science (India) cell repository. ERMS (human embryonal rhabdomyosarcoma) cells were obtained from the ATCC, USA (RD-CCL-136). BHK-21 cells were cultured and maintained in 1 × Minimum Essential Medium Eagle (MEM) (HiMedia, AL0475) supplemented with 10% Foetal Bovine Serum (FBS) (Gibco), 100 U/ml penicillin and 100 mg/ml streptomycin (HiMedia, A001A). ERMS cells were cultured in Dulbecco's Minimum Essential Medium (DMEM) (HiMedia, AL007A) with 10% FBS. The cells were grown in a humidified 5% CO_2_ incubator at 37 °C. The recombinant CHIKV-LR-5’GFP virus strain [15, 29] was used for the screening of compounds. Further validation involving both in vitro and in vivo assays was carried out using the wild-type IND-06-Guj isolate of CHIKV (GenBank: JF274082.1). Both viruses were cultured in BHK-21 cells. The virus titer was determined as plaque-forming units per milliliter (pfu/ml) in Vero cells as described before [15].

### Small molecule library

The Spectrum Collection (MicroSource Discovery Systems Inc., USA), containing biologically active and structurally diverse compounds, was used for screening the anti-CHIKV compounds. The collection contained 2560 compounds, including all the US and international drug collections. In addition, several natural and synthetic molecules were also included in the collection. The collection was available as a 10 mM DMSO solution of the compounds stored at − 80 °C.

### High-throughput drug screening

BHK-21 cells, known for their ability to efficiently replicate CHIKV, ease of culture, high growth rate, and resilience to frequent passages, were utilized for the primary antiviral screening assays, where each library compound was tested at a concentration of 10 µM (diluted in DMSO). BHK-21 cells (10,000 cells/well) were seeded in a 96-well black, flat, clear-bottom plate (Corning, 3904) in MEM supplemented with 2% FBS and incubated for 24 h at 37 °C. The cell monolayers were treated with 10 µM of each test compound and infected with CHIKV-LR-5'GFP at a multiplicity of infection (MOI) of 0.1. The plates were then incubated for 24 h at 37 °C. 0.1% DMSO, used as the vehicle for diluting the test compounds, was employed as the negative control. The cells were stained with Hoechst 33,342 (Thermo Scientific, 62249) and SYTOX Orange Nucleic Acid Stain (Invitrogen, S11368). Plate imaging was performed using the ImageXpress Micro Confocal High Content Imaging System (Molecular Devices) according to the manufacturer's instructions. Imaging was conducted using DAPI (4′,6-diamidine-2′-phenylindole dihydrochloride) to determine total cell counts, FITC (Fluorescein isothiocyanate) to identify CHIKV-infected cells, and Texas Red (SYTOX) to identify dead cells using a 10 × Plan Apo NA 0.45 objective. Data analysis was performed with the Multi-Wavelength Cell Scoring Algorithm in fast mode. The antiviral activity of each compound was determined by calculating the percentage inhibition of the GFP fluorescence. Compounds displaying > 80% fluorescence inhibition (virus replication inhibition) and > 80% viability, with respect to the DMSO vehicle-treated, CHIKV-infected control, were selected as positive hits. The secondary and tertiary screening for anti-CHIKV activity was carried out for the selected compounds in ERMS, BHK-21, and Vero cells at 1 µM and 0.5 µM concentrations, respectively.

### Selectivity index (SI) determination

ERMS cells were used to determine the selected compounds' 50% CHIKV inhibitory concentration (IC_50_). The cells were seeded at a density of 10^6^ cells/well in 96-well black flat clear-bottom plates (Corning, 3904) in DMEM supplemented with 2% FBS and incubated for 24 h at 37 °C. The cells were then treated with two-fold serial dilutions of the test compound (50 µl/well) in DMEM with 2% FBS, followed by infection with CHIKV-LR-5'GFP (MOI 5) and incubation for 30 h. The cells were then stained, and the virus replication inhibition was determined using high-content imaging, as described above. To determine the test compound’s 50% cytotoxic concentration (CC_50_), ERMS cells were seeded in 96-well plates (10^6^ cells/well) in DMEM with 2% FBS and incubated at 37 °C for 24 h. The cells were then treated with two-fold serial dilutions of the test compound (50 µl/well) and incubated for 30 h. The cells were stained with DAPI and SYTOX and analysed by high-content imaging. The percentage cell cytotoxicity was calculated from the cell viability in the test compound-treated cells compared to the DMSO-treated vehicle control. All the experiments were performed in triplicate. The IC_50_ and CC_50_ values were determined using the GraphPad Prism software, version 8.0, via nonlinear regression analysis. Each compound's selectivity index (SI) was calculated as SI = CC_50_/IC_50_. For these experiments, as well as the subsequent cell culture and animal experiments, ED (324,693) and gambogic acid (345701) were procured from Merck, and celastrol (C0869) and niclosamide (N3510) from Sigma Aldrich.

### Animal experiments

The in vivo anti-CHIKV activity of the test compounds was studied in the C57BL/6 mouse model of CHIKV infection [15]. For the experiments, the mice (10–12 weeks old, 20–25 g, either sex) were divided into three groups, each containing 10 animals. Group 1 (mock-infected) received sterile PBS, Group 2 (CHIKV-infected) received no treatment, and Group 3 (CHIKV-infected) was administered the test compound formulated in an appropriate vehicle. ED was dissolved in sterile PBS. Celastrol and gambogic acid were prepared in 10% DMSO diluted with sterile PBS, and niclosamide was dissolved in 0.5% carboxymethyl cellulose (CMC) in sterile PBS. For the virus infection, Group 2 and Group 3 mice were subcutaneously (SC) injected with a non-lethal dose of CHIKV (IND-06-Guj strain, 10^4^ pfu) in 50 µl PBS on the ventral side of both hind limbs. The CHIKV infection led to detectable blood viremia between 2–4 days post infection (pi), peaking at 2- or 3-day pi, and paw edema starting at 2–3 days pi, peaking at 6–8 days pi. The test compounds were administered intraperitoneal (IP) or SC at different times pi. The mice were monitored for two weeks with daily measurements of body weight, and paw edema using a Vernier Calliper (paw width in mm) or digital plethysmometer (paw volume in cc). The blood collected from the animals on days 2 and 3 pi was used to determine the CHIKV RNA levels, while serum was isolated to quantify the viral titers. The minimum number of animals required to achieve statistical significance was used in each experiment. Both male and female mice were included in each experimental group. In some cases, data sets from the control animals obtained for the same experimental cohort are presented across multiple figures for clarity and comparison. This was done to comply with the guidelines of CPCSEA to reduce the animal usage. Wherever such data has been reused, this is indicated in the corresponding figure legends. The Bonferroni post-hoc test, followed by a two-sided independent t-test, was used to calculate the *p* values.

### Virus binding and uptake assays

For the virus binding assay, ERMS cells were incubated with culture medium containing varying concentrations of ED for 1 h at 37 °C. In the pre-incubation protocol, following the removal of the ED-containing culture medium, the cells were incubated with CHIKV (MOI 1) in a fresh medium at 4 °C for 1 h to allow the viral adsorption. In the co-incubation protocol, the cells were incubated with CHIKV (MOI 1) in the ED-containing medium at 4 °C for 1 h to allow the viral adsorption. The cells were washed twice with PBS, lysed using RNAiso Plus (Takara, 9109) for RNA isolation, and the viral RNA levels were quantified by qRT-PCR.

For the uptake assay, ERMS cells were incubated with culture medium containing varying concentrations of ED at 37 °C for 1 h. In the pre-incubation protocol, following the removal of the ED-containing culture medium, the cells were incubated with CHIKV (MOI 1) in a fresh culture medium at 4 °C for 1 h to allow the viral adsorption. In the co-incubation protocol, the cells were incubated with CHIKV (MOI 1) in the ED-containing medium at 4 °C for 1 h to allow the viral adsorption. The cells were then washed with PBS, and the viral uptake was allowed to proceed by incubating the cells at 37 °C for 1 h in DMEM with 2% FBS. The cells were then treated with trypsin (2.5 g/L) for 1 min [15] and washed with PBS to remove any extracellular virus. The cells were washed with PBS, lysed using RNAiso Plus (Takara, 9109) for RNA isolation, and the viral RNA levels were quantified by qRT-PCR.

### Quantitative real-time PCR for CHIKV RNA

To quantify CHIKV RNA levels, total RNA was extracted from cells using the RNAiso Plus (Takara, 9109), and cDNA was prepared using 500 ng RNA, random hexamers, and the ImProm-II reverse transcription system (Promega, A3800). The relative abundance of viral RNA levels was determined by quantitative real-time PCR (qRT-PCR) using a 2 × SYBR-green reagent (Takara; RR420A) in the QuantStudio 6 Flex RT-PCR machine. The assay used a LightCycler® 96 Real-Time PCR System (Roche Life Science). *Gapdh* levels were used as the internal housekeeping control. The PCR conditions were as follows: 94 °C for 2 min (1 cycle), 94 °C for 15 s, 55 °C for 30 s, 72 °C for 1 min (40 cycles). The viral RNA levels were normalized to Gapdh and calculated by the ∆∆Ct (threshold cycle) method. All experiments had biological duplicates and were performed independently three or more times. Fold-change in the RNA level is represented as the mean ± SD of three or more independent experiments. The primers (5′−3') used in the study were as follows: GAPDH: F-TGCACCACCAACTGCTTAGC; R-GGCATGGACTGTGGTCATGAG; CHIKV: F-GGCAGTGGTCCCAGATAATTCAAG; R-GCTGTCTAGATCCACCCCATACATG; SINV: F-AAAGGATACTTTCTCCTCGC; R-TGGGCAACAGGGACCATGCA; RRV: F-GCGACGGTGGATGTCAAGGAG; R-AGCCAGCCCACCTAACCCACTG.

### Strand-specific quantitation of CHIKV RNA

The positive- and negative-sense RNA of CHIKV was quantified using a strand-specific qRT-PCR assay as described previously [15, 30]. Total RNA isolated from CHIKV-infected cells was employed for cDNA synthesis by reverse transcription. Tagged (non-viral sequence) primers PtagCHIKp and NtagCHIKn were used for cDNA synthesis from the positive- and negative-sense CHIKV RNA, respectively. As previously reported, real-time qPCR was performed using a combination of primers that bind to the non-viral tag sequence and viral strand [15]. The strand-specific RNA copy numbers were determined using the positive- and negative-sense RNA-specific standard curves. To make the standard curve, total RNA isolated from CHIKV-infected cells was used for cDNA synthesis using random hexamers. PCR was done with T7-tagged primers [15]. CHIKV nsP2 RNA was generated by in vitro transcription of the above PCR products. The RNA was quantified using a spectrophotometer and subjected to qPCR. The standard curve was plotted using Ct values obtained from a range of known RNA concentrations and the calculated copy number of strand-specific CHIKV RNA.

### Western blotting

For Western blotting, cell lysates were prepared by resuspending the cells in the pre-chilled RIPA lysis buffer (150 mM NaCl, 1 mM EDTA, 50 mM Tris, 1% Triton X-100, 1 mM PMSF) containing protease inhibitors. The lysates were incubated on ice for 2 h and then centrifuged at 13,523 × g for 15 min. The supernatant was collected, and the protein concentration was determined using the Bradford method. Electrophoresis resolved the protein sample (25 μg) on a 10% sodium dodecyl sulphate (SDS)-polyacrylamide gel. The gel was electroblotted onto a polyvinylidene fluoride (PVDF) membrane (MDI, India). Further, the membranes were blocked in 5% bovine serum albumin (BSA) prepared in Tris-buffered saline with 0.1% Tween-20. This was followed by incubation with the primary antibody overnight at 4 °C. The membranes were washed with PBS having 0.1% Tween-20 (PBST) and then incubated with HRP-conjugated secondary antibody for 1 h. The membranes were again washed with PBST, and the blots were developed using enhanced chemiluminescence (ECL) detection reagents (Cytiva, RPN2209) and visualized using ChemiDoc™ MP imaging system (Bio-Rad Labs Pvt. Ltd., India).

### Host cell protein synthesis

ERMS cells were pre-treated with varying concentrations of ED at 37 °C for 1 h, followed by the addition of 0.01 mg/mL puromycin as previously described [31]. Cells treated with DMSO served as the vehicle control. The cells were washed 12 min later with pre-warmed PBS (37 °C) and allowed to recover in the drug-free culture medium for 30 min. Cell lysates were made and subjected to western blotting using anti-puromycin monoclonal antibody (Abcam, EPR27218-173). The blots were density scanned to quantify the puromycilated proteins using the ImageJ software.

### Computational analysis

High-resolution structures of CHIKV proteins were retrieved from the RCSB Protein Data Bank (PDB) with their PDB IDs mentioned in Table 1. The three-dimensional structure of ED was obtained from the PubChem database (CID: 10219). Protein preprocessing was performed using Schrödinger’s Protein Preparation Wizard, which involved the addition of missing hydrogen atoms, assignment of bond orders, optimisation of hydrogen-bonding networks, and energy minimisation using the OPLS2005 force field. The ligand ED was prepared using the LigPrep module to generate its protonated, low-energy conformations, followed by desalting and stereoisomer generation where applicable. The SiteMap analysis was performed using Schrödinger to identify the potential ligand binding site(s) on the protein’s surface. A receptor grid was generated around the predicted active site of the helicase, focusing on conserved motifs essential for the ATPase activity. Molecular docking was carried out to evaluate the binding of ED to CHIKV proteins using Schrödinger’s Glide module in extra-precision (XP) mode [15]. Flexible docking of the ligand was performed to explore potential binding orientations. The optimal binding pose was selected based on the GlideScore, interaction analysis, and visual inspection to identify the key interacting residues. Binding affinity (expressed in kcal/mol) was estimated using the docking score. Post-docking binding free energy (ΔG_bind_) was calculated using the Prime Molecular Mechanics with Generalised Born Surface Area (MMGBSA), incorporating solvation and entropy contributions via single-point energy calculations based on the Generalised Born Surface Area (GBSA) model. To evaluate the dynamic stability of ED at the potential binding site on CHIKV nsP2, molecular dynamics (MD) simulations were conducted using the Desmond module for 100 ns following standard relaxation protocols. The trajectory analysis was performed to assess interaction stability throughout the simulation, which included monitoring the root mean square deviation (RMSD) of the protein–ligand complex. The key binding residues were identified using the ligand interaction fingerprinting and hydrogen bond analysis tools.

**Table 1:** In silico docking of ED with CHIKV proteins

Protein	PDB ID	Docking score	References
nsP1	7DOP	− 1.457	[32]
nsP1	7X01	− 4.645	[32]
nsP2 helicase	6JIM	− 5.399	[33]
nsP2 Protease	3TRK	− 5.607	[34]
nsP3	3GPG	− 4.402	[35, 36]
nsP4	7Y38	− 1.427	[37]
Capsid	5H23	− 3.016	[38]
E1–E2–E3 complex	3N41	− 4.280	[36, 39]
E1–E2–E3 complex	3N43	− 4.119	[36, 39]

### Expression and purification of CHIKV nsP2 protein

The full-length CHIKV nsP2 protein, as well as its helicase and protease domains, were expressed and purified as described previously [15]. Briefly, a modified pET14b expression plasmid encoding the gene of interest and an N-terminal His_6_-SUMO tag was used to transform *Escherichia coli* Rosetta (DE3) cells. Cultures were grown in LB to an OD_600_ of 0.6–0.8 and induced with 0.5 mM isopropyl β-d-1-thiogalactopyranoside (IPTG) at 18 °C for 16 h. Cells were harvested and lysed in a buffer containing 20 mM HEPES pH 7.5, 500 mM NaCl, 5% glycerol, and 2 mM β-mercaptoethanol. The lysate was clarified and applied to a Ni–NTA column (Cytiva), followed by elution using an imidazole gradient. His_6_-SUMO tag was removed using PreScission protease and the sample was dialyzed overnight at 4 °C. The protein was concentrated and subjected to size-exclusion chromatography using a Superdex 200 16/600 (Cytiva) column in 20 mM HEPES pH 7.5, 300 mM NaCl, 5% glycerol, and 0.5 mM DTT. The purity was confirmed by SDS PAGE.

### Isothermal titration calorimetry (ITC)

The ED binding interactions of CHIKV nsP2 full-length protein and its truncated domains were studied by ITC using the MicroCal ITC-200 machine (MicroCal, USA). The proteins and ED were prepared in a buffer containing 20 mM HEPES (pH 7.5), 250 mM NaCl, 5% glycerol, and 1% DMSO. The calorimetric cell was loaded with 10 µM protein, while 100 µM ED was loaded into the syringe for titration into the reaction cell. The titration consisted of an initial 0.4 µl injection, followed by 18 injections of 2 µl at 150 s intervals, with an initial delay of 60 s. The titration was conducted at 25 °C with a reference power of 10 µcal/s and a stirring speed of 750 rpm. The raw ITC data and titration plots were analysed using Malvern's Origin 7.0 Microcal-ITC200 analysis software, employing a one-site binding model to calculate the thermodynamic parameters, including the dissociation constant (*K*_d_), and enthalpy change (ΔH). The thermodynamic changes due to ED dilution were negligible when the buffer was titrated against ED and vice versa. The binding interactions were characterised by significant changes in differential power (DP) in µcal/sec and binding enthalpy (ΔH) profiles, as shown in the isotherms, with the solid lines representing the best least-squares fitting to the data.

### Microscale thermophoresis (MST)

The ED binding to the CHIKV nsP2 protein was studied by MST on the Monolith NT.115 instrument (NanoTemper) as per the manufacturer's instructions. For the MST experiments, 10 µM protein was labelled on lysine residues with RED-NHS fluorescent dye using the 2nd Generation Monolith Protein Labelling RED-NHS kit (NanoTemper). The dye contains a reactive NHS-ester group that forms a covalent bond with the primary amines, such as lysine residues, by reacting with them. For binding affinity, the labelled protein (1 µM) was incubated with two-fold serial dilutions of ED or a 28-mer synthetic RNA substrate (5′-AAAAAAAAAAAACCAGGCGACAUCAGCG-3′) [40, 41] and loaded into the Monolith NT.115 capillaries to obtain the binding curve. The data was collected using the Monolith NT.115 device with 60% (RED) LED power and analysed using MO Affinity Analysis v2.3 software. For the binding affinity analysis, ligand-dependent changes in temperature-related intensity change (TRIC) were plotted as Fnorm values against ligand concentration in a dose–response curve. The Fnorm values were expressed in parts per thousand (‰). For each trace, the Fnorm value was determined by dividing F1 by F0, where F1 represents the fluorescence value measured in the heated state, and F0 corresponds to the fluorescence value measured in the cold state before the IR laser is activated. The MST data showed an affinity curve with a well-defined bound and unbound states. The dissociation constants (*K*_d_) for the interaction of CHIKV nsP2 and its individual helicase and protease domains with ED were determined using the MO affinity analysis software (Nanotemper technologies).

### Site-directed mutagenesis

The polymerase chain reaction (PCR) was used for the site-directed mutagenesis employing overlapping primers on the expression plasmid containing the CHIKV nsP2 cDNA. The overlapping primers were designed according to the desired nucleotide changes in the coding region of the protein. The PCR was carried out using Phusion polymerase (NEB, M0530S) and the reaction mix was digested with Dpn I (NEB, R0176S) to digest the methylated parent plasmid (the template). The resultant product DNA was purified and used to transform the *E. coli* DH5α competent cells. The cells were grown at 37 °C, and the plasmid was isolated. The cDNA was subjected to nucleotide sequencing to verify the sequence with the desired mutation.

### Helicase assay

To investigate the effect of ED on the RNA unwinding activity of CHIKV nsP2, a helicase assay was performed following the protocol described previously [40, 41]. The assay utilised an Alexa Fluor 488-labelled 28-mer synthetic single-stranded RNA (Alexa488-ssRNA, 5′-AAAAAAAAAAAACCAGGCGACAUCAGCG-3′) and an unlabelled 16-mer ssRNA oligonucleotide (5′-CGCUGAUGUCGCCUGG-3′) to generate a dsRNA helicase substrate with a 12-base 5′ overhang. To prepare the dsRNA substrate, the labelled and unlabelled RNA oligonucleotides were mixed at a 1:1.1 molar ratio in a buffer containing 10 mM HEPES (pH 7.2) and 20 mM KCl. To facilitate annealing, the RNA mixture was heated to 95 °C for 1 min in a thermal cycler, followed by gradual cooling at a rate of 1 °C per min until reaching 22 °C. The optimized reaction mixture for the unwinding assay consisted of the strand-displacement assay buffer (40 mM HEPES, pH 7.5, 2 mM dithiothreitol (DTT), and 12 mM NaCl), 1 μM purified CHIKV nsP2 protein or its helicase and protease domains, 50 nM dsRNA substrate, 800 nM unlabelled RNA trap (5'-CCAGGCGACAUCAGCG-3'), 20 U RNaseOUT inhibitor (Thermo Fisher Scientific, 10,777,019), and varying concentrations of ED, and Withaferin A (a CHIKV protease inhibitor) as a negative control. The reaction mixture was incubated at 26 °C for 20 min in a thermal cycler to facilitate the formation of the protein-RNA complex. Subsequently, a 3.5 mM ATP-magnesium acetate mixture was added, followed by incubation at 37 °C for 120 min. The reactions were then terminated by adding a stop solution containing 100 mM Tris–HCl, pH 7.5, 0.1% bromophenol blue, 1% SDS, 50 mM EDTA, and 50% glycerol. The separation of ssRNA and dsRNA was carried out by electrophoresis on a 15% nondenaturing polyacrylamide gel at 4 °C. The RNA bands were visualized using a Typhoon imager (Cytiva), and their intensities were quantified using the ImageJ software. Denatured ssRNA (prepared by heating dsRNA at 95 °C for 5 min, followed by rapid cooling at 4 °C) was used as a control for dsRNA. BSA protein (1 μM) was used as a negative control to assess the unwinding activity of nsP2.

### ATPase assay

The colorimetric ATPase assay measured the release of inorganic phosphate from ATP hydrolysis as described previously [42]. The assay was done in a 96-well plate using the EnzChek Phosphate Assay Kit (E6646, Invitrogen). The reaction buffer contained 20 mM HEPES (pH 7.0), 2 mM MgCl₂, 10 mM KCl, 1 mM DTT, and 250 mM NaCl. For the assay, the 2-amino-6-mercapto-7-methylpurine riboside substrate (MESG, 200 µM), 20X reaction buffer, purine nucleoside phosphorylase (PNP, 1 unit/mL), 50 nM CHIKV nsP2, and 1 or 10 µM ED were mixed, and the reaction mixture was incubated on ice for 10 min. The reaction was initiated by adding ATP (250 µM), and absorbance was recorded continuously at 360 nm at 10-s intervals. A standard curve was generated using K₂PO₄ provided with the kit as the inorganic phosphate source, and the nmol of Pi released was plotted against time.

### CHIKV nsP2 protease assay

The proteolytic activity of the CHIKV nsP2 protease was determined using a FRET-based approach [43, 44]. The assay was performed in vitro using the purified CHIKV nsP2 protein and the FRET-based octapeptide substrate {DABCYL}-RAGG↓YIFSS-{Glu(EDANS)}-NH2 (Biolink), representing the nsP3/4 site [15, 45]. For the assay, 1 μM CHIKV nsP2 was incubated with different concentrations of ED in the assay buffer (20 mM Bis–Tris-Propane, pH 8) for 30 min at 25 °C. Following incubation, the nsP2-ED mixture was added to a Nunc 96-well black plate (Thermo Fisher Scientific, 137101) with 25 μM peptide substrate in the assay buffer and the fluorescence was measured at 43 s intervals using a multimode plate reader SpectraMax i3x at an excitation wavelength of 340 nm and emission wavelength of 490 nm. A substrate control reaction measured the auto-fluorescence generated by just the substrate. Withanone was used at 10 μM concentration as a negative control, and withaferin A at 10 μM concentration as a positive control for the protease activity inhibition assay [15]. All enzyme reactions were performed in duplicates and two independent experiments. The average fluorescence of each sample was calculated and plotted in the graph.

### Cy5 labelling of RNA

Three hundred pmol of 28-mer single-stranded synthetic RNA (5′-AAAAAAAAAAAACCAGG CGACAUCAGCG-3′) was fluorescently labelled at the 3′-end with Cy5 using 0.6 U/μL T4 RNA ligase and 20 μM pCp-Cy5 (Jena Bioscience, NU-1706-CY5) in buffer (New England Biolabs, M0204S) supplemented with 1 mM ATP and 20% DMSO and incubated at 16 °C for 15 h. Following ligation, the Cy5-labelled RNA was purified using the RNeasy Mini Kit (Qiagen, 74,104) and eluted in nuclease-free water [46].

### Alphavirus antiviral assay

ERMS cells were seeded in 12-well plates and infected with Ross River virus (RRV) (T48 strain) or Sindbis virus (SINV) (AR339 strain) at an MOI of 1. After virus infection for 1 h at 37 °C, the inoculum was removed, cells were washed with PBS, and fresh complete medium containing the indicated concentrations of ED was added. At 6 h pi, culture supernatants were collected for viral titration, and cells were harvested for RNA extraction. Viral titers in the supernatants were determined by plaque assay. Total RNA was extracted from infected cells using the RNAiso Plus kit (Takara, 9109), followed by RT-qPCR using virus-specific primers. Viral RNA levels were normalized to *Gapdh*, and relative RNA levels were determined using the comparative Ct method.

### Statistical analysis

The animal edema and weight data were analyzed by the Bonferroni post-hoc test, followed by a two-sided independent t-test used to calculate the *p* values. In all other experiments, Student’s t-test was used for the *p* value calculation. The *p* values are indicated in the figures with stars as *p < 0.05, **p < 0.01, ***p < 0.001, ****p < 0.0001, ns = not significant.

## Results

### Screening of the Spectrum Collection for anti-CHIKV compounds

BHK-21 cells were used for the screening assay, for which various conditions, such as the cell density, viral-infective dose as the multiplicity of infection (MOI) and assay endpoint, were standardised. Ribavirin was used as a positive control since it has been shown to inhibit CHIKV in vitro and in vivo [15]. To ensure a reliable conclusion, validation of the assay was done by studying the quality parameters; the signal-to-noise (S/N) ratio was ~ 50, the coefficient of variation (CV) was < 1.9%, and the Z’ factor was 0.91.

**Fig. 1:**
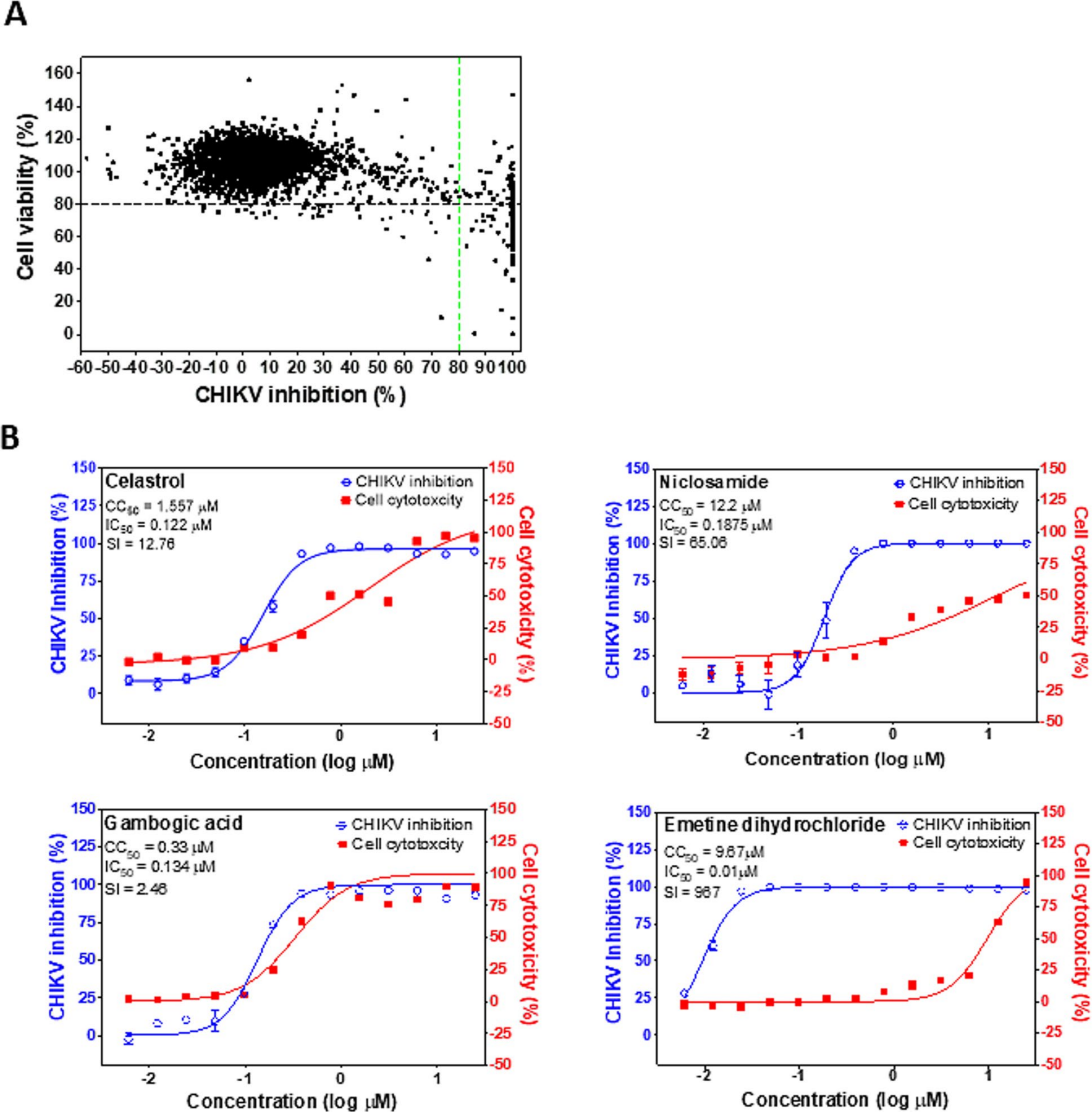
Screening of the Spectrum Collection for CHIKV antiviral activity. **A** For the primary screening, BHK-21 cells were seeded in a 96-well plate and infected with CHIKV-GFP at 0.1 MOI for 20 h in the presence of DMSO (vehicle control) or the test compounds at 10 µM concentration. The figures show the percentage of cell viability and CHIKV replication inhibition. **B** ERMS cells were infected with CHIKV-GFP (MOI 1) and treated with different concentrations of the test compounds. The dose–response curve demonstrating cell toxicity and CHIKV inhibition at different concentrations at 24 h pi is shown

In the primary screening of the Spectrum Collection in BHK-21 cells, 164 compounds showed > 80% cell viability and > 80% CHIKV inhibition at 10 µM concentration (Fig. 1A, Table S1). In the secondary screening, these compounds were screened at 1 µM concentration in ERMS (Fig. S1), BHK-21, and Vero cells, where 28 compounds showed > 80% cell viability and > 80% CHIKV inhibition in any of the three cell lines used (Table S1). In the tertiary screening, these 28 compounds were further screened for anti-CHIKV activity at 0.5 and 0.1 µM concentrations (Table S2). Four compounds, celastrol, niclosamide, gambogic acid, and ED showed anti-CHIKV activity (> 80% virus inhibition) in all three cell lines at 0.5 µM concentration, and these were studied further. The CC_50_ and IC_50_ of these compounds were determined in ERMS cells to calculate the selectivity index (SI) (Fig. 1B). The SI value for celastrol was 123, for niclosamide it was 65, and for gambogic acid it was 2. ED had a remarkably high SI value of 967.

### Anti-CHIKV efficacy of the selected compounds in mice

**Fig. 2:**
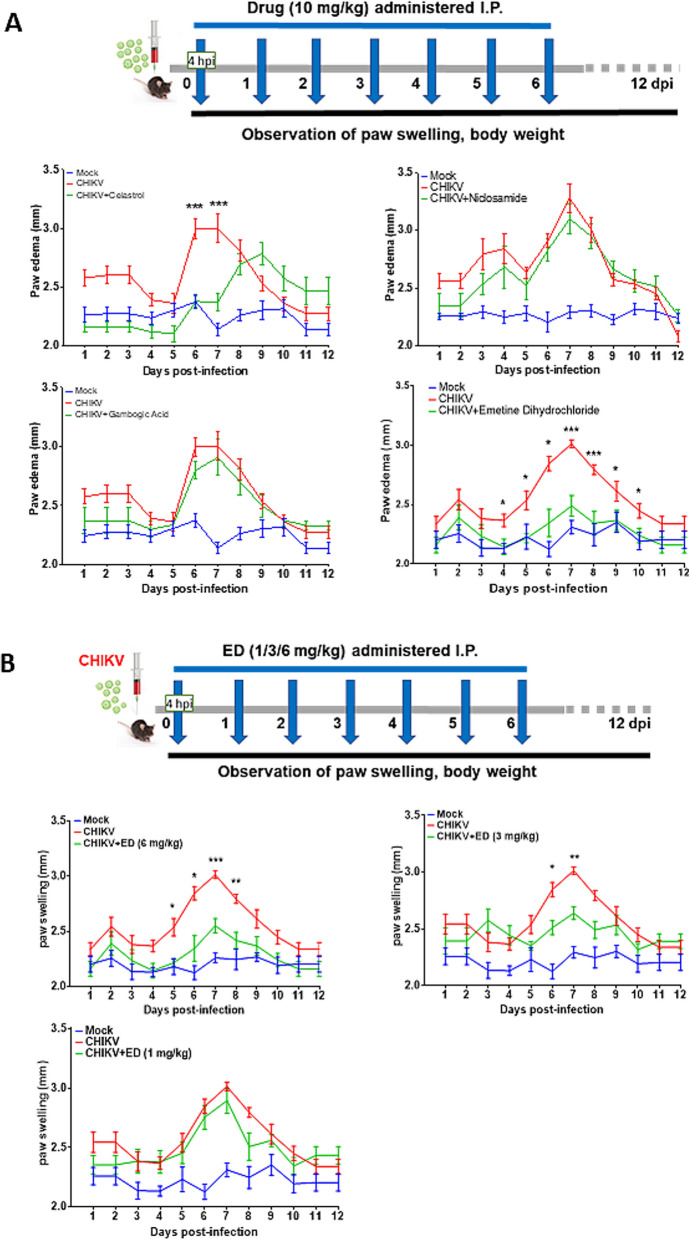
Anti-CHIKV efficacy of the selected compounds in a mouse model of disease. **A** C57BL/6 mice of 12 weeks of age were mock-infected (n = 6) or infected SC (n = 7–8 in each group) with 10^4^ pfu of CHIKV and treated with vehicle alone or the indicated drug compound (10 mg/kg) given IP once a day. The first dose of the compound was delivered 4 h pi. The mice were followed for 12 days, and the paw edema was measured daily as paw width/thickness using a Vernier Calliper. A line graph demonstrating the paw edema on different days pi is presented. **B** C57BL/6 mice at 12 weeks of age were mock-infected or infected SC with 10^4^ pfu of CHIKV and treated with vehicle alone or the indicated dose of ED (1, 3, or 6 mg/kg) given IP once a day. The first dose of the compound was delivered 4 h pi. The mice were followed for 12 days, and the paw edema was measured daily as paw width/thickness using a Vernier Calliper

The antiviral potential of celastrol, niclosamide, gambogic acid, and ED was tested in our previously published mouse model of CHIKV infection in adult C57BL/6 mice, where CHIKV-infected mice show viremia and footpad edema (Fig. S2), which is self-resolving [15]. Mice inoculated SC in the footpad with CHIKV began to show footpad edema at 4 to 5-day pi, with maximum edema recorded at 6 to 7-day pi, after which the edema was seen to self-resolve, reaching normal levels by 10 to 12-day pi. CHIKV-infected mice were treated with the above compounds at a dose of 10 mg/kg/d delivered IP, 4 h after virus infection (Fig. 2A). Treatment of CHIKV-infected mice with niclosamide or gambogic acid did not affect virus-induced footpad edema. Celastrol treatment delayed the footpad edema without affecting its extent. ED-treated CHIKV-infected mice, however, showed no edema (*p* < 0.001). The footpad edema measurements of the ED-treated CHIKV-infected mice were statistically not different from those in the mock-infected mice.

The recommended human dose of ED is 1 mg/kg/d [47] which translates to a dose of 12 mg/kg/d for a mouse [48]. Since there are some concerns of toxicity at this human dose [47, 49] we tested in mice half the human dose, which comes to 6 mg/kg/d. While the 6 mg/kg/d and 3 mg/kg/d doses effectively reduced the footpad edema, the dose of 1 mg/kg/d was not effective when delivered IP (Fig. 2B).

**Fig. 3:**
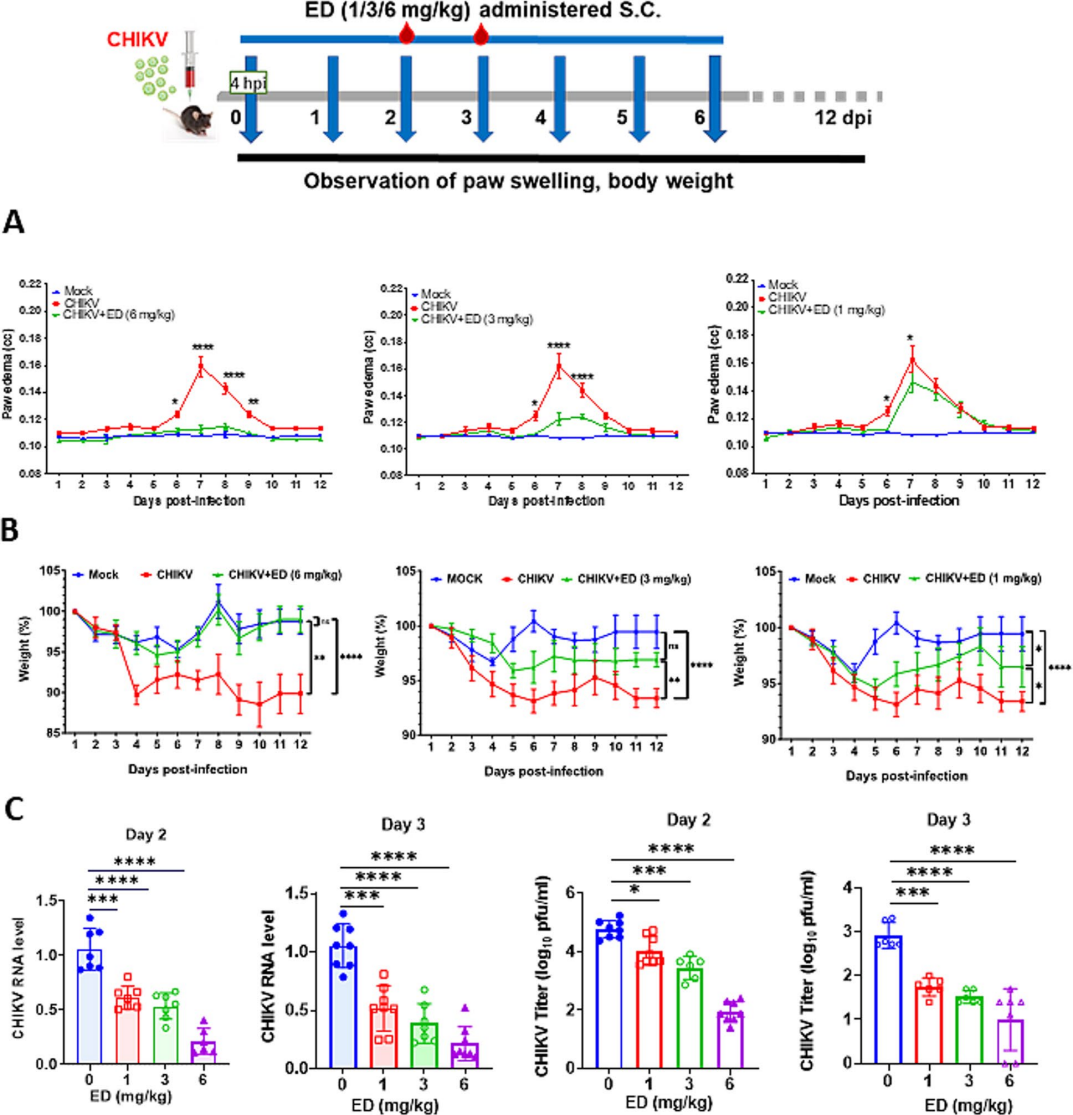
Anti-CHIKV efficacy of ED in a mouse model of disease. C57BL/6 mice of 12 weeks of age were mock-infected (n = 6) or infected SC (n = 8 in each group) with 10^4^ pfu of CHIKV and treated with vehicle alone or the indicated dose of ED given SC once a day. The first dose of ED was delivered 4 h pi. The mice were followed for 12 days, and the paw edema was measured daily using a digital plethysmometer. Blood was collected on 2- and 3-day pi (d pi). The paw edema of the mice on different d pi is presented in **A** while **B** shows the mouse weight on different d pi. The relative CHIKV RNA levels (compared to the infected but no ED-treatment control) and viral titers in the mice blood at 2- and 3-d pi are presented in **C**. All three treatments were studied simultaneously in a single experiment. The three panels have been made for clarity only and present the same controls: the CHIK-infected untreated mice (red lines) and the mock-infected mice (blue lines)

In humans, a SC drug delivery is more practical than the IP one. Accordingly, we studied the efficacy of ED delivered SC in CHIKV-infected mice (Fig. 3A). A dose-dependent effect was seen on the virus-induced footpad edema in CHIKV-infected mice treated with graded doses of ED. CHIKV-infected mice treated with the 6 mg/kg/d dose of ED showed no footpad edema. Mice treated with the 3 mg/kg/d dose of ED showed significantly reduced footpad edema compared to the control. However, ED at the 1 mg/kg/d dose did not reduce the virus-induced footpad edema in CHIKV-infected mice. Similarly, there was no weight loss in the CHIKV-infected mice treated with a 6 mg/kg/d dose of ED, whereas weight loss was not fully recovered in CHIKV-infected mice treated with ED at a dose of 3 mg/kg/d or 1 mg/kg/d (Fig. 3B). The antiviral effect of ED in CHIKV-infected mice was reflected in the significantly reduced CHIKV viremia (Fig. 3C). Here also, a dose–response was seen in the reduced CHIKV titer in mice treated with the graded doses of ED, with the highest reduction recorded in mice treated with the 6 mg/kg/d dose.

### Antiviral potential of ED against CHIKV in mice

**Fig. 4:**
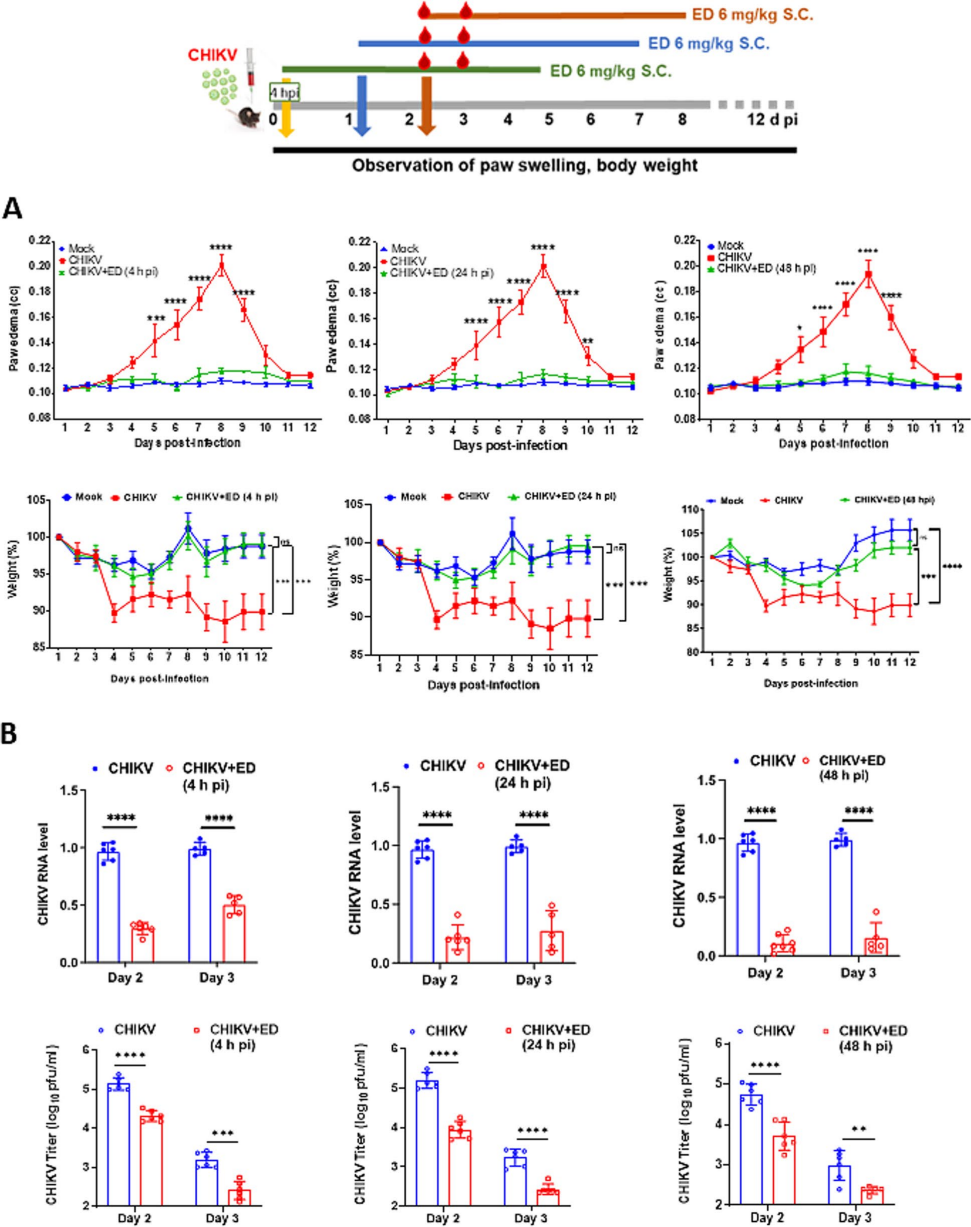
Antiviral efficacy of delayed ED treatment in a mouse model of chikungunya disease. C57BL/6 mice at 12 weeks of age were mock-infected (n = 7) or infected SC (n = 8 in each group) with 10^4^ pfu of CHIKV and treated with vehicle alone or ED (6 mg/kg) given SC once a day. The first dose of ED was delivered at 4 h pi (0 d pi), 24 h pi (1 d pi), or 48 h pi (2 d pi) as indicated. The mice were followed for 12 days, and the paw edema (**A**) and mouse weight (**B**) were measured daily. Blood was drawn from the animals on 2 and 3 d pi (3–4 h after drug administration). The relative CHIKV RNA levels (compared to the no ED-treatment control) and viral titers in the blood are presented in **C**. All three treatments were studied simultaneously in a single experiment. The three panels have been made for clarity only and present the same controls: the CHIK-infected untreated mice (red lines) and the mock-infected mice (blue lines)

To test the antiviral potential of ED to treat CHIKV patients, CHIKV-infected mice were treated with ED (6 mg/kg/d) administered SC at different times pi. ED treatment was effective in suppressing the virus-induced footpad edema when delivered at 4 h pi, 24 h pi, and as late as 48 h pi (Fig. 4A). A similar effect of ED was seen on the mouse weight (Fig. 4A). Again, ED treatment led to a reduction in viremia in CHIKV-infected animals (Fig. 4B). Drug treatment beyond 48 h pi was not investigated.

### CHIKV replication kinetics in ERMS cells in the presence of ED

**Fig. 5:**
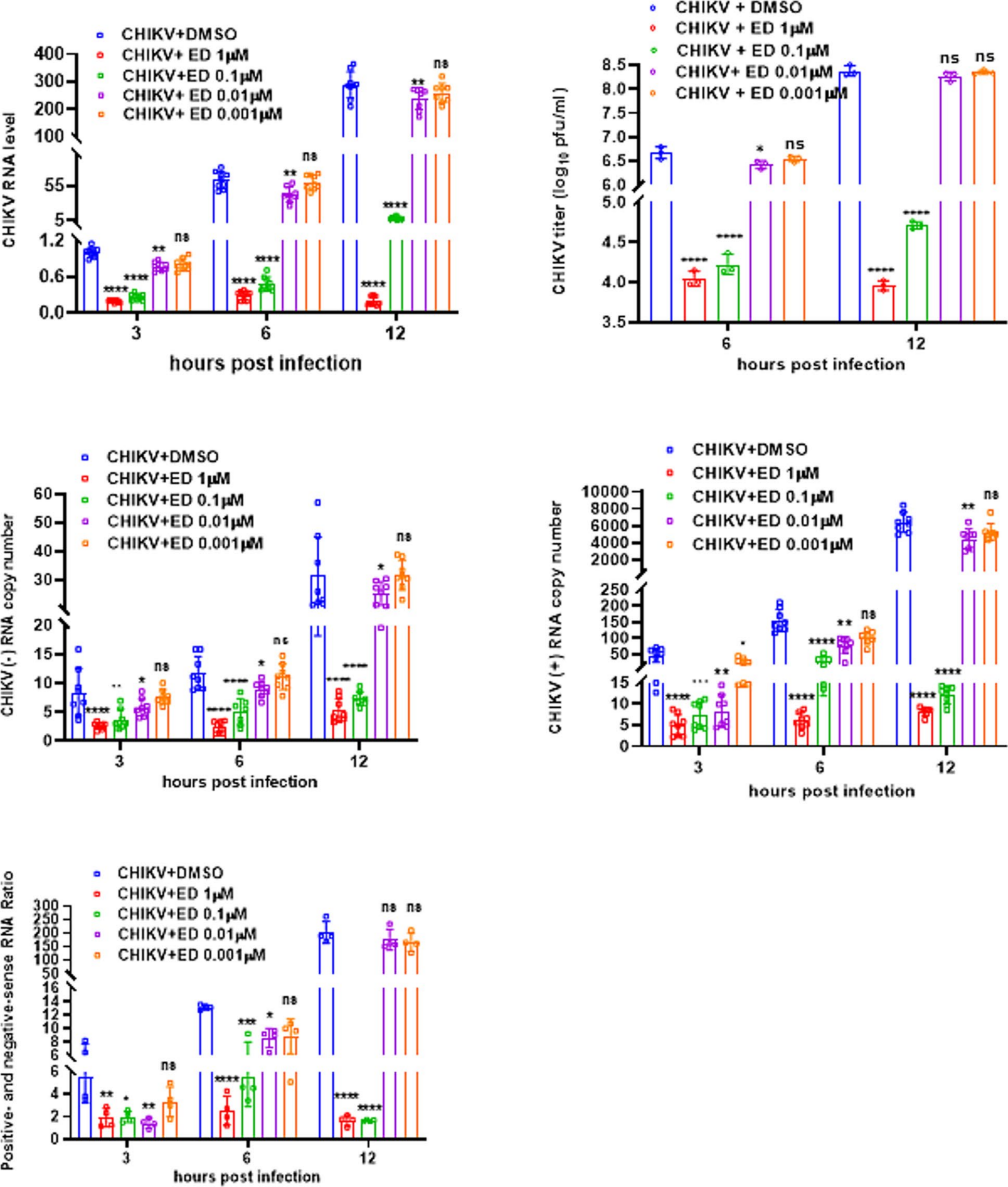
CHIKV replication kinetics in ERMS cells in the presence of ED. ERMS cells were infected with CHIKV (1 MOI) and incubated with different concentrations of ED. Control CHIKV-infected cells were incubated with DMSO. The cells and culture supernatants were harvested at different times pi for the extraction of the total RNA and determination of the viral titers, respectively. qRT-PCR was used to determine CHIKV RNA levels and positive- and negative-sense CHIKV genome copy numbers. Relative viral RNA levels are shown in the top left panel, where *Gapdh* was used as an internal control. The RNA level in the control at 3 h pi was taken as one. CHIKV titers determined by plaque assay are shown in the top right panel. The negative- and positive-sense CHIKV genomic RNA copy numbers are shown in the middle panel. The bottom panel shows the ratio of positive- and negative-sense RNAs. Data from the control (DMSO-treated) cells were compared with those treated with ED at different time points. Data representing the mean ± SD from three independent experiments, each performed in triplicate, are shown

To understand the mechanism of ED’s antiviral action, the CHIKV replication kinetics were studied in the presence of various ED concentrations. CHIKV genome replication, as determined by CHIKV RNA levels, was significantly suppressed in the ED-treated CHIKV-infected cells as early as 3 h pi and this suppression continued until 12 h pi (Fig. 5). Viral RNA levels were lower in cells treated with ED at concentrations as low as 0.01 µM. Viral titers increased with time in CHIKV-infected control cells. However, these were significantly lower in the presence of ED concentrations as low as 0.01 µM and 0.1 µM at 6 and 12 h pi, respectively. Importantly, viral RNA replication and viral titers in the presence of varying ED concentrations showed a dose response effect.

In the CHIKV-infected control cells, the negative-sense CHIKV RNA could be detected at 3 h pi, and its copy number continued to increase till 12 h pi (Fig. 5). In CHIKV-infected ED-treated cells, however, the negative-sense CHIKV RNA levels were significantly lower in the presence of ED concentrations as low as 0.01 µM (Fig. 5). Similarly, the positive-sense RNA levels were also significantly lower in the presence of ED concentration as low as 0.01 µM. While the ratio of positive- and negative-sense RNA showed a sharp increase in CHIKV-infected control cells, this was not seen in the cells treated with ED concentrations as low as 0.1 µM (Fig. 5), indicating a significant inhibition of CHIKV RNA synthesis.

### ED affects early events during CHIKV replication

**Fig. 6:**
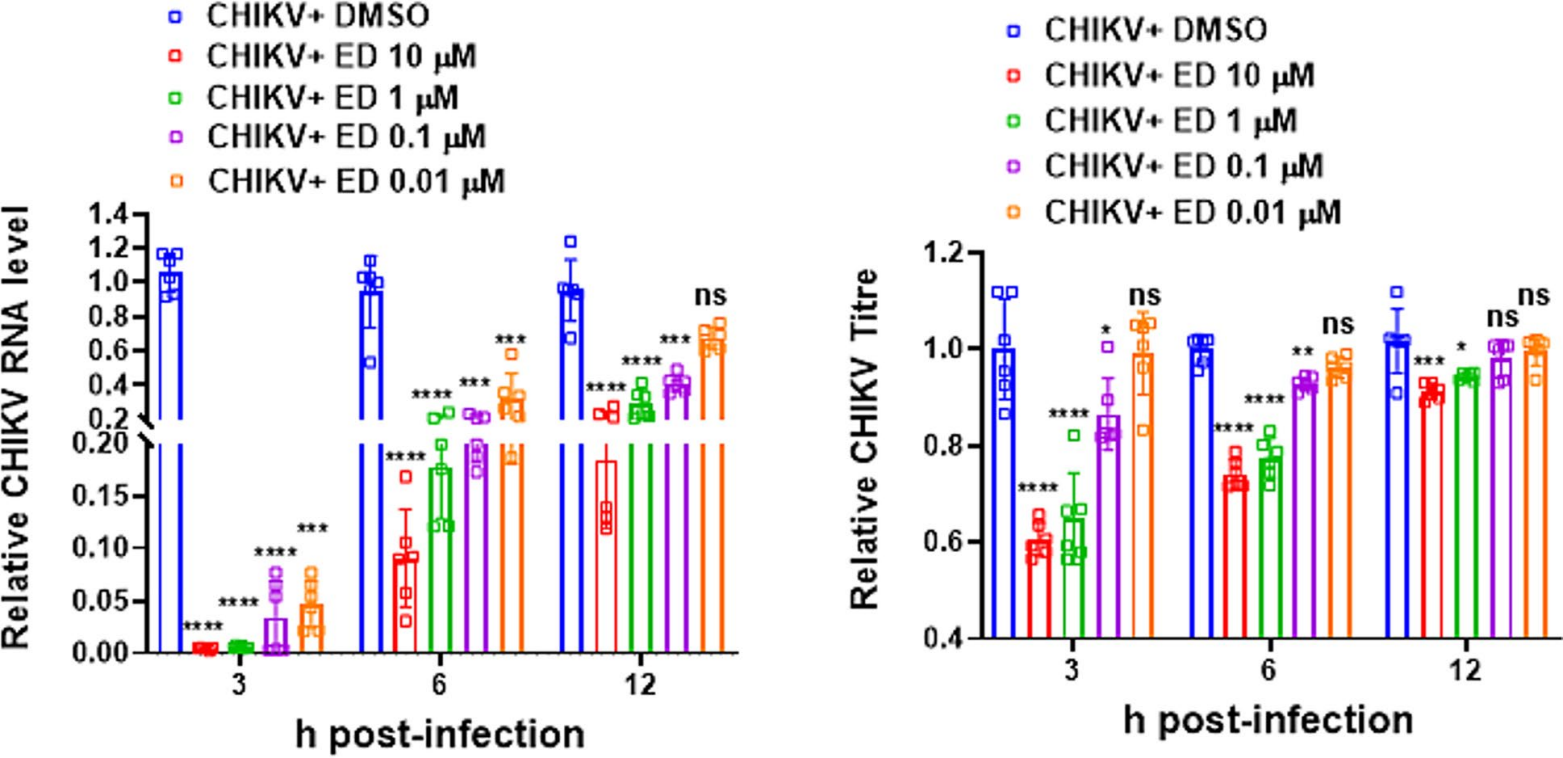
Time-of-addition assay. ERMS cells were infected with CHIKV (MOI 1) and ED was added at different concentrations to the culture medium at 3, 6, or 12 h pi. The cells and the culture supernatants were collected 24 h pi to determine the relative viral RNA titers in ED-treated and untreated control sample where only DMSO was added. Relative CHIKV RNA levels were quantified by qRT-PCR, normalized to *Gapdh* as an internal control, and expressed relative to DMSO-treated infected controls. Infectious viral titers in culture supernatants were determined by plaque assay. Relative viral titers were calculated by normalizing the titers from ED-treated samples to the DMSO-treated infected control. Data representing the mean ± SD from three independent experiments, each performed in triplicate, are shown

A time-of-addition assay was done to understand which step/s of the CHIKV replication might be affected by ED (Fig. 6). CHIKV RNA levels and viral titers in CHIKV-infected cells treated with ED at different times pi were studied. The data show a significant inhibition of viral RNA synthesis and titers when ED was added as early as 3 h pi. The inhibitory effect was reduced when ED was added at later time points. These data suggest a post-entry mode of drug action affecting early replication events.

### ED inhibits viral protein synthesis in CHIKV-infected cells

**Fig. 7:**
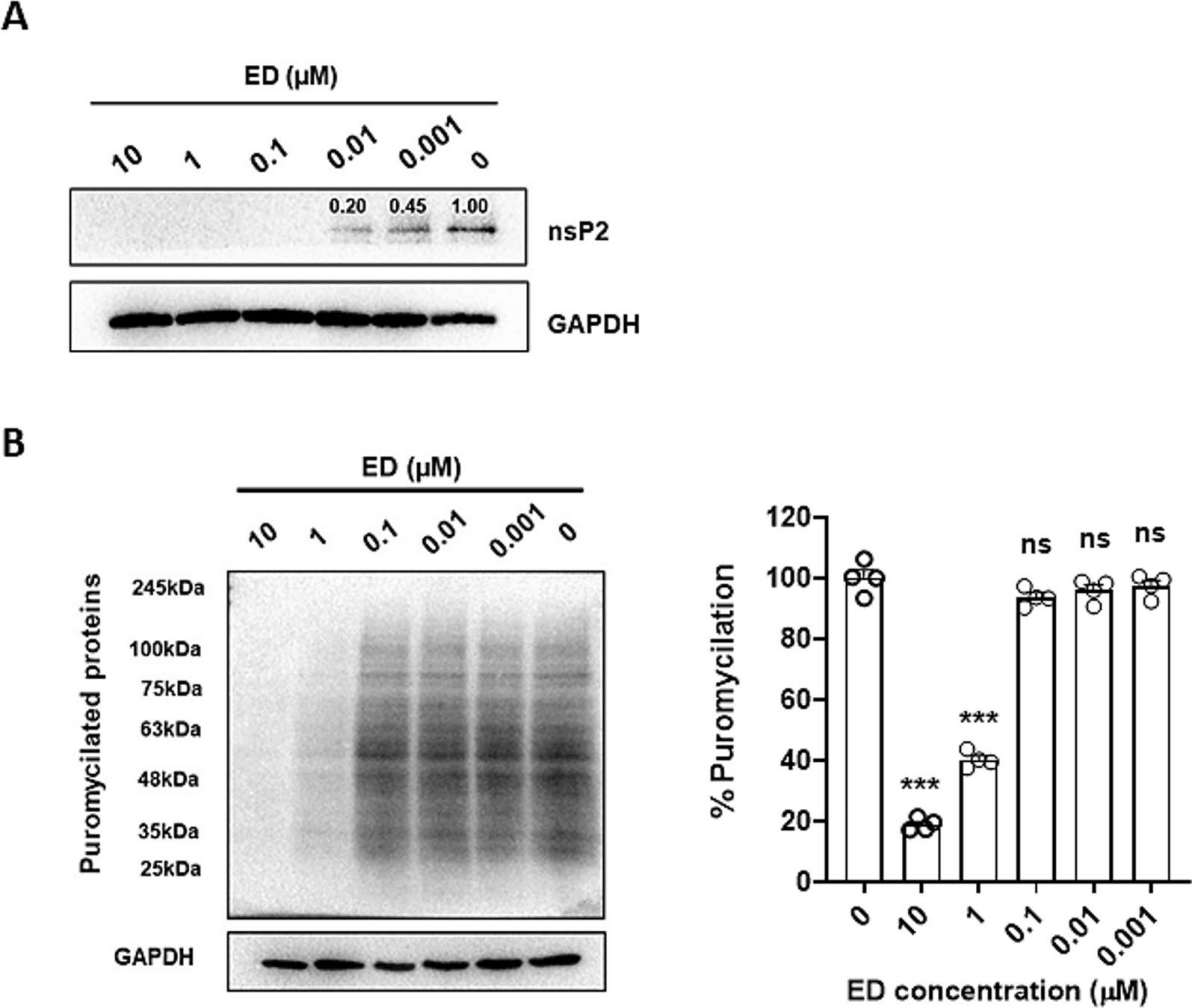
ED inhibits CHIKV protein synthesis. **A** ERMS cells were infected with CHIKV (MOI 1) and treated with different concentrations of ED, while control CHIKV-infected cells were treated with DMSO only. Cell lysates were collected at 12 h pi and western blotted with the CHIKV nsP2 antibody. GAPDH was used as the loading control. Relative band intensity was quantified using the ImageJ software and displayed over the bands. **B** ERMS cells were incubated in DMEM containing 2% FBS with varying concentrations of ED for 1 h at 37 °C, followed by the addition of puromycin at a final concentration of 0.01 mg/mL. Cells treated with culture medium supplemented with DMSO but without ED served as the control. The cells were washed 12 min later with pre-warmed PBS (37 °C) and allowed to recover in DMEM containing 2% FBS for 30 min. The cell lysates were western blotted with anti-puromycin monoclonal antibody. A representative western blot is shown at the left. GAPDH was used as the loading control. The lane intensities, measured by the ImageJ software, relative to the control, are shown at the right. Data represent mean ± SD from two independent experiments, each performed in duplicates

The reduced negative-sense RNA levels in the presence of ED early during the infection and the time-of-addition assay suggested that the drug inhibited the early step/s of the CHIKV life cycle. During the early phase of infection, the RNA genome of CHIKV is translated into a single polyprotein, which is subsequently processed to produce seven non-structural proteins. These proteins are required for the synthesis of the negative-sense genomic intermediate RNA that acts as a template for genome replication. We therefore studied viral protein synthesis in CHIKV-infected cells in the presence of increasing ED concentrations (Fig. 7A). We followed the synthesis of the nsP2 protein for which a good quality antibody was available in the lab. The nsP2 levels are representative of the other viral proteins as they are made in equimolar amounts from the cleavage of a polyprotein [50]. Western blotting of CHIKV-infected ERMS cells in the presence of graded doses of ED showed inhibition of the viral protein synthesis in the presence of ED at a dose as low as 0.1 µM. However, protein synthesis inhibition waned at further lower doses of ED.

ED inhibits protein synthesis in eukaryotic cells by blocking the ribosome movement along the mRNA during elongation [51]. We therefore studied whether ED inhibited cellular protein synthesis at concentrations where CHIKV protein synthesis was inhibited (Fig. 7B). We performed puromycin labelling of host cell proteins to track the formation of puromycylated peptides in the presence or absence of ED. Puromycin integrates into the nascent polypeptide chain, prematurely terminating translation and generating puromycylated peptides. These peptides can be detected using western blotting with an anti-puromycin antibody. The host cell protein synthesis was almost completely inhibited at 10 µM ED concentration, and it was partially inhibited at 1 µM ED concentration. However, at 0.1 μM or lower ED concentration, host cell protein synthesis was largely unaffected.

These experiments showed that the viral protein synthesis in CHIKV-infected cells was inhibited at 0.1 µM of ED, whereas at this concentration the host cell protein synthesis was not significantly affected. These data indicated that the CHIKV protein synthesis inhibition may not be due to the general ribosome-targeted effect of ED on translation [52–54], but due to an alternate mechanism.

### ED binds to the CHIKV nsP2 protein in silico

To decipher the mechanism of the ED-mediated CHIKV replication inhibition, we examined if ED could bind with any of the CHIKV proteins. The crystal or cryo-EM structures of CHIKV proteins are available in the PDB (Table 1). CHIKV nsP2 is a multifunctional non-structural protein with a C-terminal cysteine protease and N-terminal helicase activity-containing domains. Thus, for the nsP2 protein, the crystal structures of the individual domains are available [55] and these were used in our study. The study used the structures of the structural proteins E1, E2, and E3 in a complex, the way they exist in the virions [39]. We studied the in silico binding of ED to different CHIKV proteins using the Schrödinger computational platform (Table 1). These data show that ED was docking with the helicase and protease domains of the nsP2 protein with the most negative ΔG values, corresponding to a more thermodynamically favourable binding, suggesting ED’s potential binding with the CHIKV nsP2 protein.

### ED binds to the CHIKV nsP2 helicase domain

**Fig. 8:**
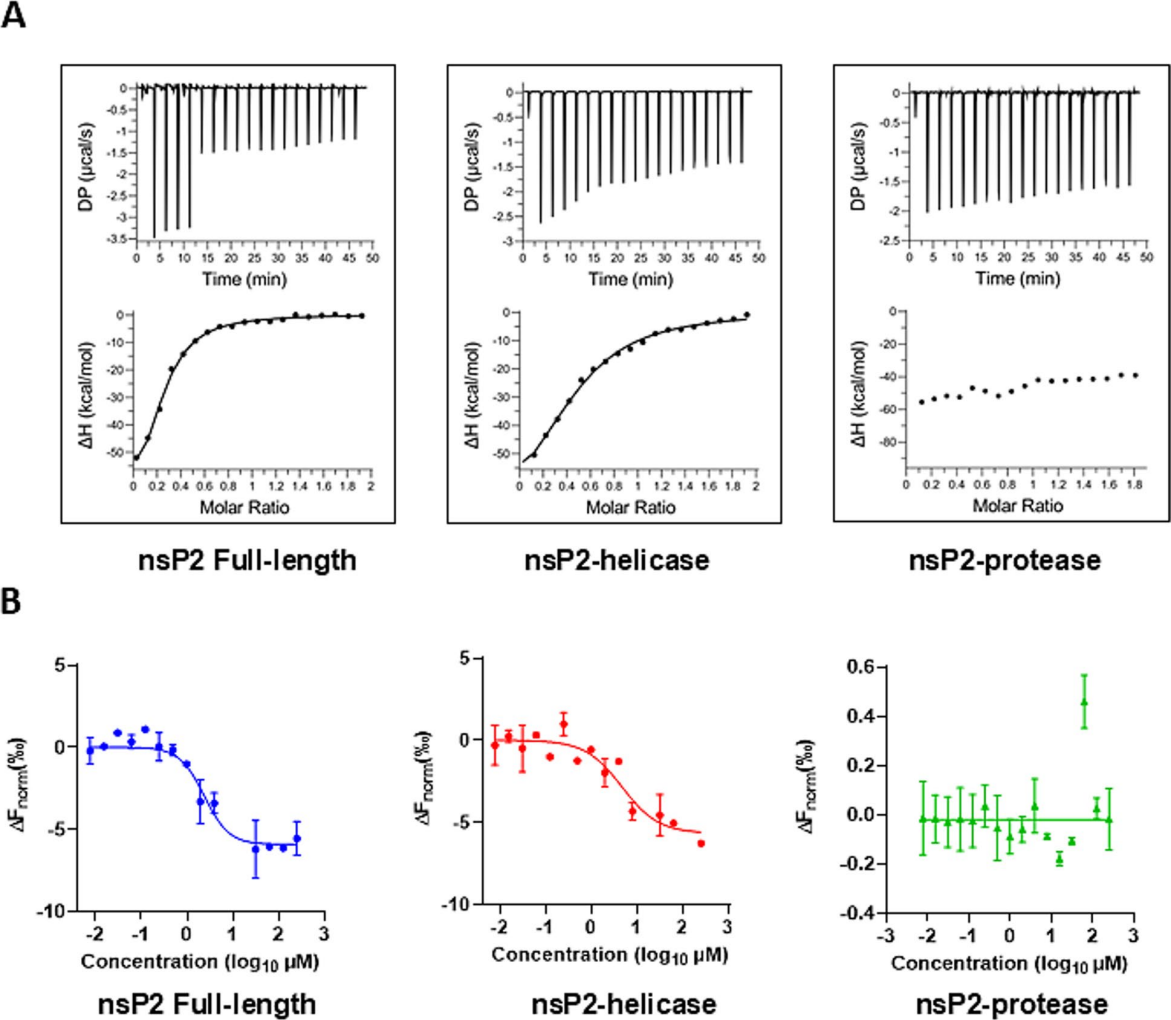
ED binds to the CHIKV nsP2 helicase domain. **A** ITC was used to study the binding of the CHIKV nsP2 protein or its helicase and protease domains with ED. The binding reaction was titrated using 10 µM protein and 100 µM ED. The thermograms (top) and fitted binding isotherms (bottom) for the binding of the nsP2 full-length protein and its truncated domains to ED are shown. Malvern’s Origin 7.0 Microcal-ITC200 analysis software was used to obtain the dissociation constant (*K*_d_) and thermodynamic parameter ΔH. **B** MST was used to study the binding of the labelled CHIKV nsP2 protein (1 μM) or its helicase and protease domains with different concentrations of ED. For the binding affinity analysis, ligand-dependent changes in temperature-related intensity change (TRIC) are plotted as Fnorm values against the ED concentrations in a dose–response curve. The Fnorm values are plotted as parts per thousand (‰)

To experimentally validate the above predictions, the binding of the CHIKV nsP2 protein with ED was studied using ITC (Fig. 8A). ED was found to bind to the full-length nsP2 protein in a dose-dependent manner, with a *K*_d_ value of 1.2 µM. We then evaluated the ED binding of the helicase and protease domains of the nsP2 protein. Here, ED was seen binding with the helicase domain in a dose-dependent manner, although the binding affinity of 2.56 µM was lower than that seen with the full-length nsP2 protein. ED showed no binding with the protease domain.

The ITC data was further validated using the MST method (Fig. 8B). A dose-dependent binding of ED to full-length nsP2 was seen with a *K*_d_ value of 1.5 µM. As seen in the ITC experiment, here also ED bound to the helicase domain with a lower affinity, with a *K*_d_ of 3.06 µM, while ED showed no binding with the protease domain.

### ED inhibits the nsP2 helicase activity

**Fig. 9:**
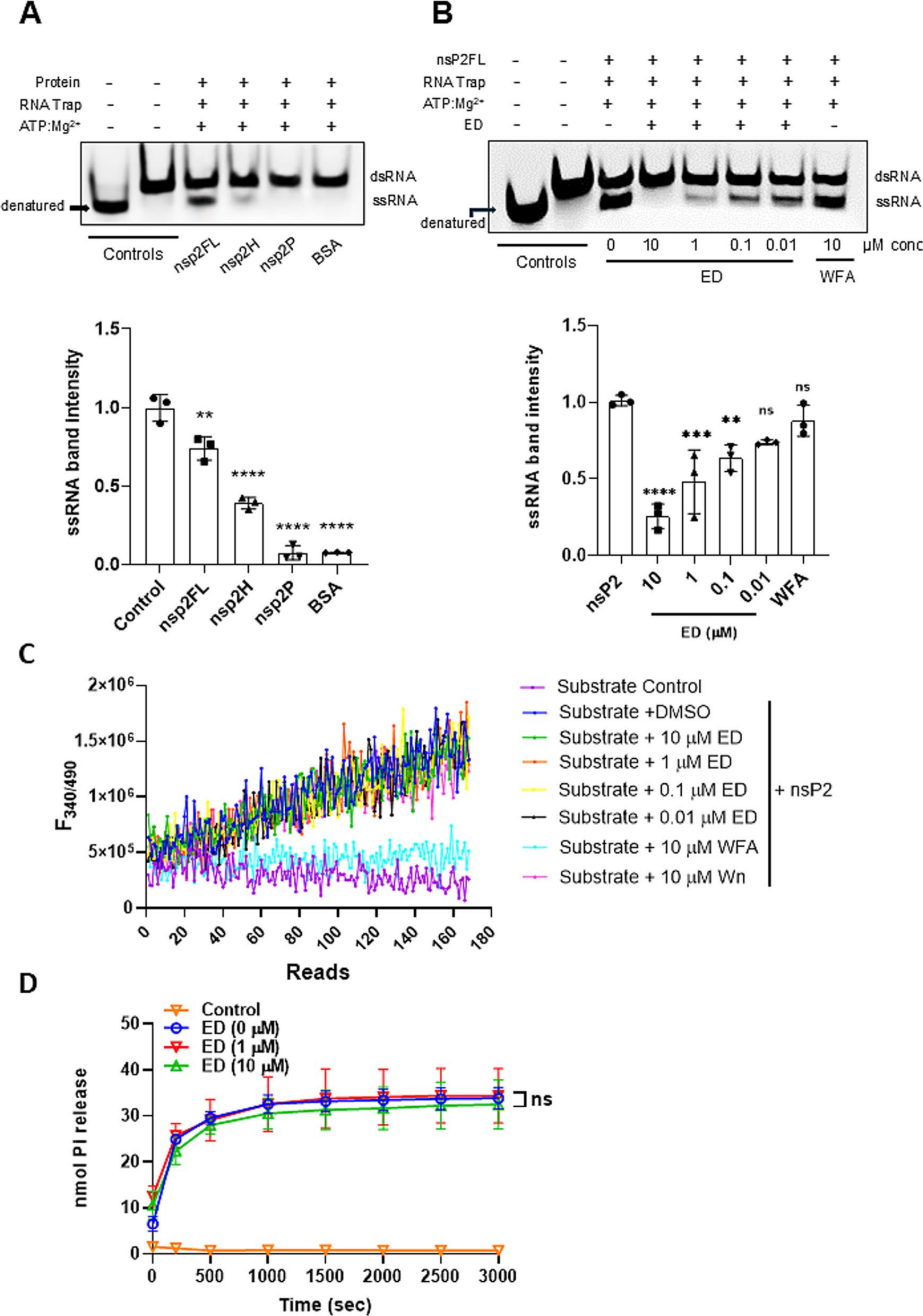
ED inhibits CHIKV nsP2 RNA helicase activity. **A** The RNA helicase activity of the full-length nsP2 (nsP2FL) and its helicase (nsP2H) and protease (nsP2P) domain proteins was studied using the Alexa488-dsRNA28/16 substrate. BSA was used as a negative control. **B** The effect of ED on the RNA helicase activity of the nsP2FL protein was studied using the Alexa488-dsRNA28/16 substrate. WFA was used as a negative control. Representative gel pictures are shown in the top panel. The mean ± SD of relative band intensities from 3 experiments, quantified using the ImageJ software, are provided in the bottom panels. **C** A FRET-based protease assay was used to study the nsP2 protease activity. A real-time profile of the proteolytic assay is presented using 25 μM fluorogenic peptide substrate, 1 μM nsP2 protein, with different concentrations of ED. WFA, a known inhibitor of nsP2 protease, and withanone (Wn), known to not inhibit the nsP2 protease activity, were used as the controls. The fluorescence was monitored every 43 s. The background fluorescence of the substrate peptide was monitored without the enzyme and is shown as substrate control. **D** A colorimetric ATPase assay was performed using 50 nM CHIKV nsP2 as the enzyme and 250 µM ATP as the substrate. The line diagram illustrates the ATPase activity of CHIKV nsP2 in the presence of two different concentrations of ED. Data are represented as mean ± SD from three independent experiments. The statistical difference between the different treatment groups, as determined by two-way ANOVA, was not significant (ns)

The CHIKV nsP2 helicase activity was examined using an Alexa488-tagged synthetic dsRNA. The full-length nsP2 was able to efficiently unwind the dsRNA to produce the Alexa488-tagged ssRNA (Fig. 9A). The nsP2 helicase domain also showed unwinding activity, although it was less efficient than the full-length nsP2. The nsP2 protease domain, however, did not show any helicase activity. BSA used as a negative control showed no detectable unwinding activity. The effect of ED on nsP2 helicase activity was studied using this assay. ED inhibited the helicase activity of the nsP2 protein in a dose-dependent manner (Fig. 9B). Significant inhibition of helicase activity was seen at 0.1 µM ED concentration, at which the virus RNA and protein synthesis and viral replication were also inhibited. In a control reaction, Withaferin A (WFA), known to inhibit the nsP2 protease activity [15] did not inhibit the nsP2 helicase activity. We also tested if ED inhibited the nsP2 protease activity using a protease assay validated previously in our lab [15]. ED concentrations as high as 10 µM did not inhibit the nsP2 protease activity in vitro (Fig. 9C).

The N-terminal domain of CHIKV nsP2 functions as an ATP-dependent RNA helicase (SF1 superfamily), where ATP hydrolysis provides energy for 5′–3′ dsRNA unwinding [40, 41]. Thus, CHIKV nsP2 helicase activity may also be impaired by ED inhibiting the ATPase activity. To examine this, ATPase activity of CHIKV nsP2 was assayed in the presence of ED (Fig. 9D). CHIKV nsP2 exhibited robust ATPase activity, reflected by steady phosphate accumulation over time. Importantly, ED at 1 and 10 µM concentrations did not inhibit the ATPase activity, whereas the helicase activity was significantly inhibited at these ED concentrations (Fig. 9B). No inhibitor specifically targeting CHIKV nsP2 ATPase activity is commercially available that could be used as a control. These data suggest that ED may be specifically targeting the helicase activity without inhibiting the ATPase activity.

### Characterization of the ED binding site on the nsP2 helicase

**Fig. 10:**
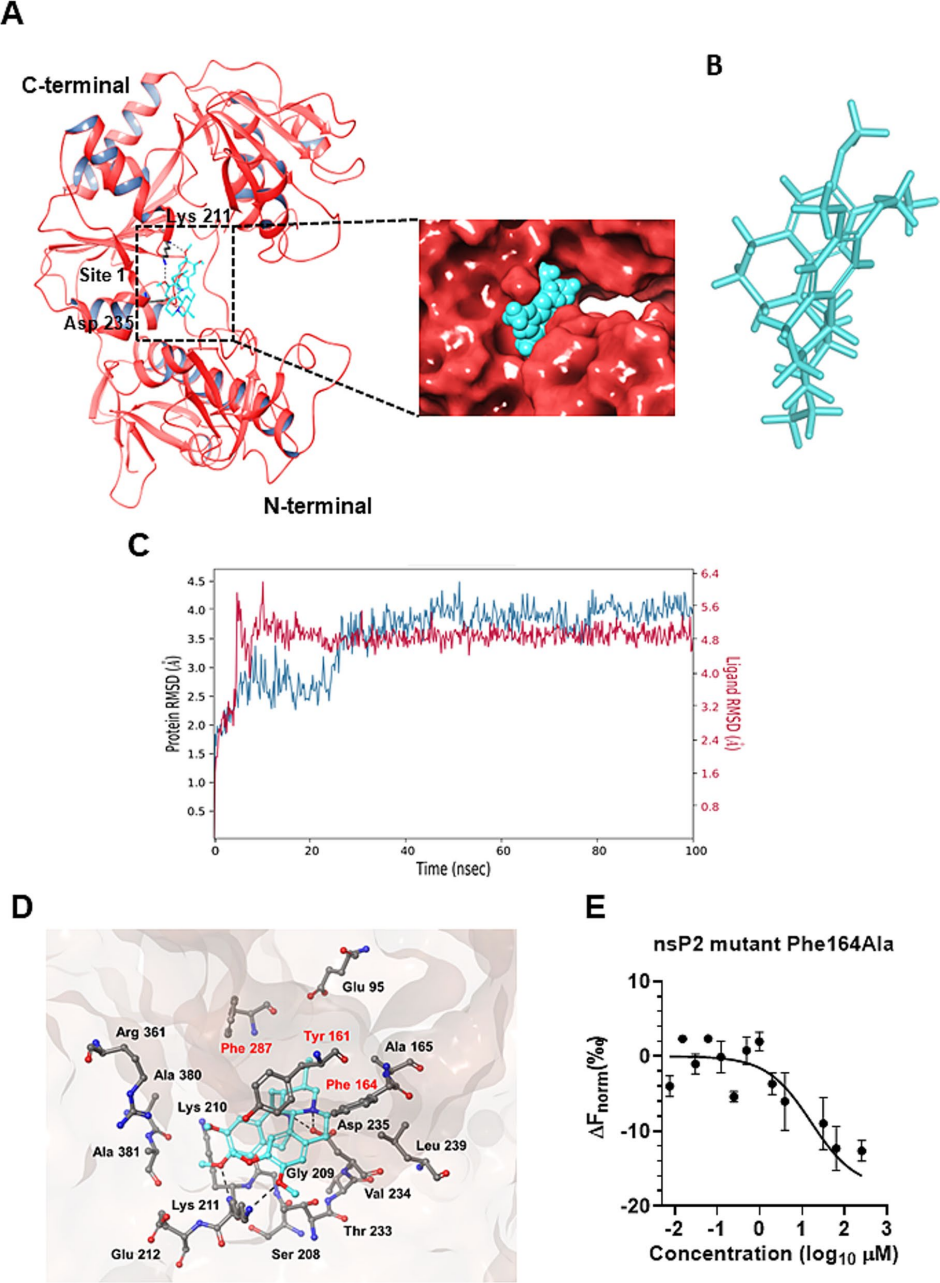
ED binding to CHIKV nsP2 helicase in silico. **A** The structure of the CHIKV nsP2 helicase protein (PDB: 6JIM) with docked ligand (ED) is shown. The protein is rendered in a cartoon representation and coloured in red, while ED is shown in a stick representation and coloured in cyan. The binding of ED to Site-1 is shown. The hydrogen bonding residues of nsP2 are represented in stick with grey. The inset shows the fitting of ED into the respective pocket; the protein is rendered in surface view in red colour, and ED is shown in a vdW surface representation. **B** The structure of the ligand (ED) is shown in the stick representation. **C** The RMSD plot of the CHIKV nsP2 protein and ED interaction at Site-1 from the MD analysis is shown. **D** The interacting residues of nsP2 with ED within the Site-1 within 4.0 Å distance are shown. The interaction between the ligand and the protein is shown with black dotted lines. The protein and the ligand are shown in the stick representation. Amino acids involved in substrate binding are indicated in red text. **E** MST was used to study the binding of 1 μM labelled Phe164Ala mutant of CHIKV nsP2 protein with different concentrations of ED. For the binding affinity analysis, ligand-dependent changes in temperature-related intensity change (TRIC) are plotted as Fnorm values against ED concentrations in a dose–response curve. The Fnorm values are plotted as parts per thousand (‰)

We studied in silico the ED binding with the nsP2 helicase by the SiteMap analysis, which predicted five potential binding sites in the helicase domain of nsP2 (Table S3). Site-1, with the best site score of 1.019, was selected for molecular docking with ED (Fig. 10A). ED binds to Site-1 with a docking score of − 5.399 kcal/mol. In addition, thermodynamic calculations were performed on the docked poses using the Prime MMGBSA program of the Schrodinger suite, which showed ED's strong binding to Site-1 with a binding free energy (ΔG_bind_) value of − 61.77 kcal/mol. The docking analysis revealed that ED was positioned in a well-defined orientation within Site-1, forming stable interactions with the key residues of the nsP2 protein helicase active site [33].

Further, to check the conformational stability of the nsP2 helicase-ED complex, the MD simulations were carried out for 100 ns. The RMSD analysis of the MD trajectories showed that ED was stable at Site-1, and its binding stabilised the overall structure of the nsP2 protein until 100 ns (Fig. 10C). The MD simulation outcomes corroborated the docking findings that ED could bind to Site-1 on CHIKV nsP2 helicase domain.

The interaction fingerprinting identified the key amino acid residues contributing to the most stable state obtained from the MD simulation (Fig. 10D). Nine amino acids of the nsP2 helicase domain line the ED within the 4.0 Å distance. The major contributors included positively charged (Lys210, Lys211), negatively charged (Asp235), hydrophobic (Phe286, Phe287, Phe164), non-polar (Ala381), and polar (Thr261, Tyr161) amino acids (Fig. 7D). Among these, Lys211 and Asp235 formed hydrogen bonds with ED, while Lys210 showed pi-pi stacking interactions with ED. These data indicate that ED has the potential to interact with the key residues (Tyr161, Phe164, and Phe287) of the substrate binding site/active site of CHIKV nsP2 helicase described previously by others [33, 56].

To validate the ED binding site predicted in silico*,* a Phe164Ala mutant of nsP2 was made, and its binding with ED was evaluated by MST. While full-length nsP2 bound to ED with a *K*_d_ value of 1.5 µM (Fig. 8), the mutant showed a significantly reduced *K*_d_ value of 29.40 µM (Fig. 10E), indicating a significant reduction in binding of the mutant protein with ED compared to the wild type nsP2. These data show a role for the amino acid Phe164 in the nsP2 interaction with ED.

### ED binds around the RNA-binding site of the nsP2 helicase

**Fig. 11:**
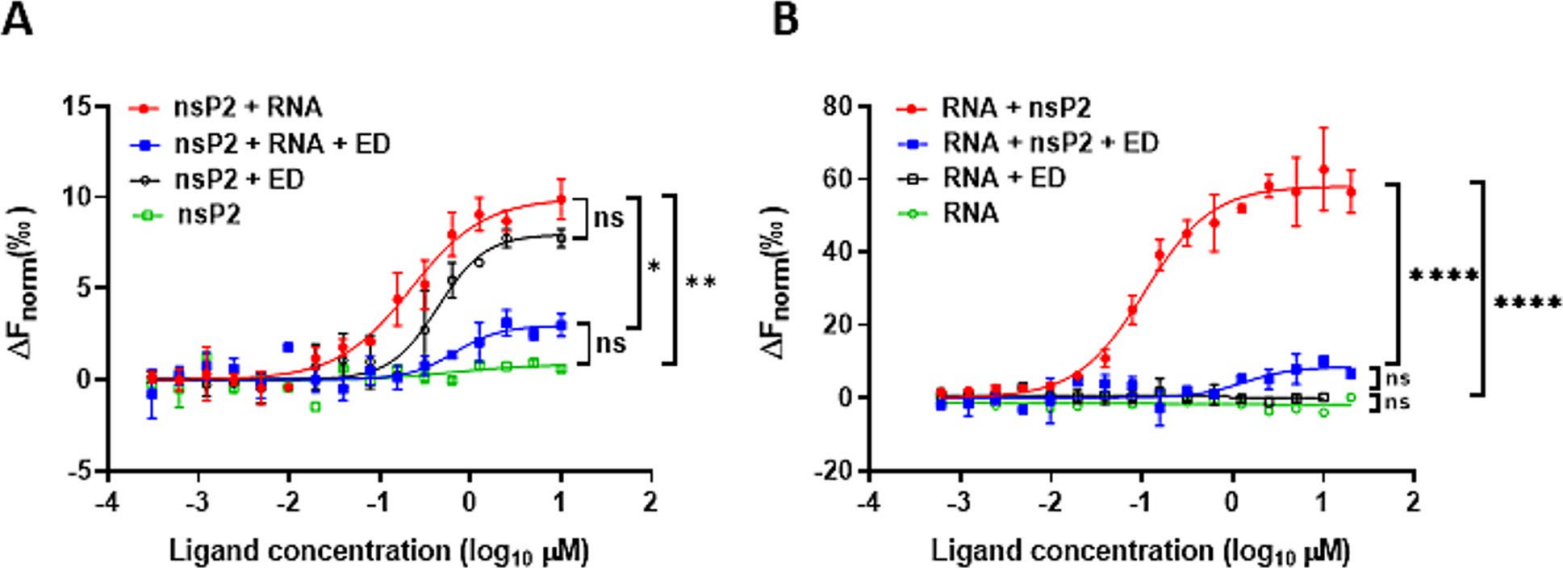
ED competes for the RNA-binding site of the CHIKV nsP2 protein. **A** MST was performed using Red-NHS dye labelled full-length nsP2 protein (50 nM) titrated with increasing concentrations of the ligands 28-mer single-stranded RNA, ED, or the RNA-ED complex. **B** MST was performed using Cy5-labeled 28-mer single-stranded RNA (10 nM) titrated with increasing concentrations of the ligands nsP2 protein, ED, or the nsP2-ED complex. Normalized fluorescence changes (ΔFnorm) are plotted against the ligand concentration

The data above showed that ED inhibited the nsP2 helicase activity. The nsP2 helicase activity requires a stable RNA binding as a prerequisite for the ATP-driven unwinding [33, 57, 58]. The MST experiment showed that nsP2 bound to the RNA substrate with a *K*_d_ of 297 nM, whereas nsP2 bound to ED with a lower affinity, having a *K*_d_ of 583 nM. The nsP2 protein-ED complex binding with the RNA substrate had a drastically lower *K*_d_ of 1.12 µM; the binding curve was distinctly closer to the free protein trace, indicating that ED was competing for binding to the RNA binding site on the nsP2 protein (Fig. 11A). This inhibition of RNA-nsP2 protein binding by ED was further validated by studying the concentration-dependent binding of Cy5-labelled RNA to the nsP2 protein (Fig. 11B). The RNA–protein binding had a *K*_d_ value of 256 nM, which was markedly reduced to 29 µM in the presence of ED, while ED alone showed no detectable interaction with the RNA. The significant loss of RNA–nsP2 binding in the presence of ED indicates that ED binds at or around the RNA-binding site of the nsP2 protein, competitively interfering with the RNA–protein interaction. These data validate the in silico studies that predicted the ED binding site in or around the same location as the RNA substrate binding site on CHIKV nsP2 protein.

### ED inhibits CHIKV uptake

**Fig. 12:**
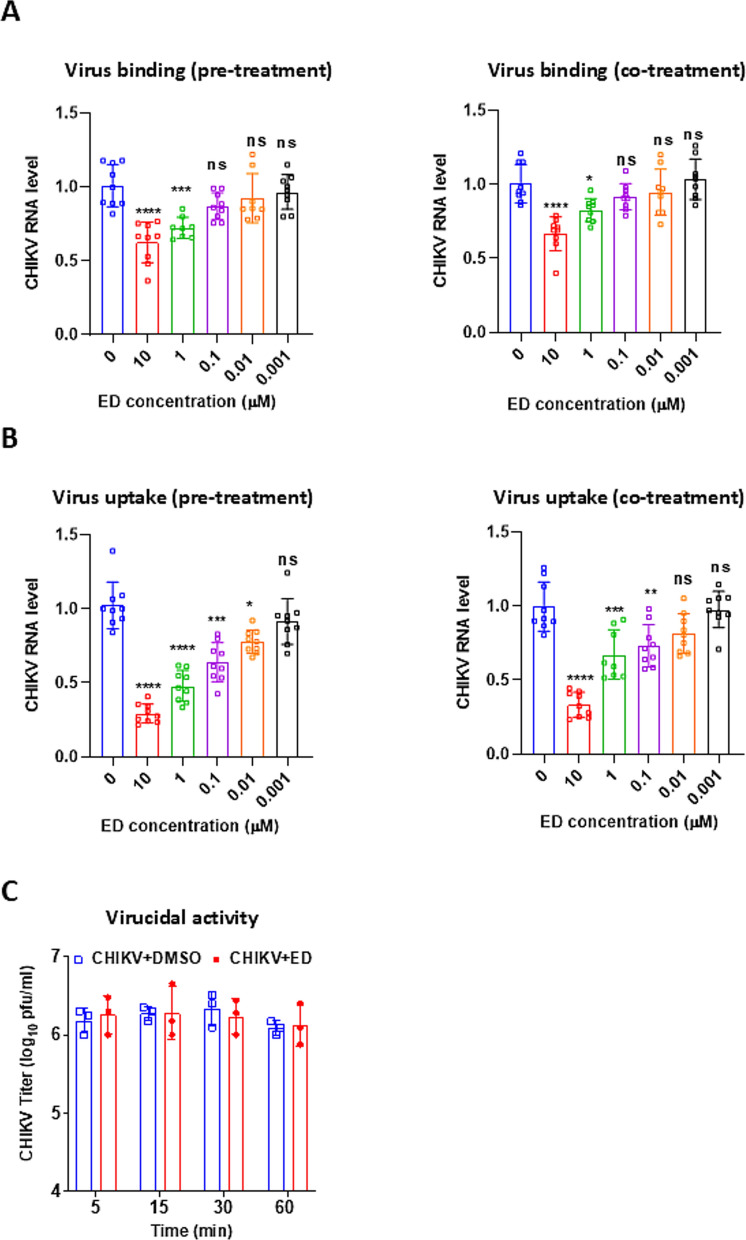
CHIKV binding and uptake in host cells in the presence of ED. **A** To study the virus binding, ERMS cells were incubated with CHIKV (MOI 1) in the presence of different concentrations of ED under pre-treatment or co-treatment protocol. **B** To study the virus uptake, ERMS cells were incubated with CHIKV (MOI 1) in the presence of different concentrations of ED under pre-treatment or co-treatment protocol. Following a PBS wash, the cells were harvested for RNA isolation. Relative CHIKV RNA levels are shown, where *Gapdh* was used as the internal control. The viral RNA levels in the control (DMSO-treated) cells were compared with those treated with ED. **C** For the virucidal assay, CHIKV in DMEM was incubated with 20 µM ED or DMSO (control) for 1 h at 37 ºC. The virus titers were then determined by plaque assay. Viral titers in the control were compared with those in the presence of ED; no significant difference was found

Other events that can affect virus replication early during the CHIKV life cycle include the virus binding to the host cell and its subsequent uptake. CHIKV binding to ERMS cells was studied under drug pre-treatment as well as co-treatment conditions. In both cases, CHIKV binding was not affected at 0.1 µM ED concentration (Fig. 12A) at which the virus's RNA and protein synthesis, and viral replication were inhibited. However, virus uptake showed ~ 30% inhibition at this concentration of ED (Fig. 12B). The inhibition of virus binding and uptake at higher ED concentrations may be related to inhibition of host protein synthesis. The virucidal activity of ED was also ruled out when CHIKV titers were not affected when the virus was incubated with 20 µM ED (Fig. 12C).

### ED inhibits the replication of different alphaviruses

**Fig. 13:**
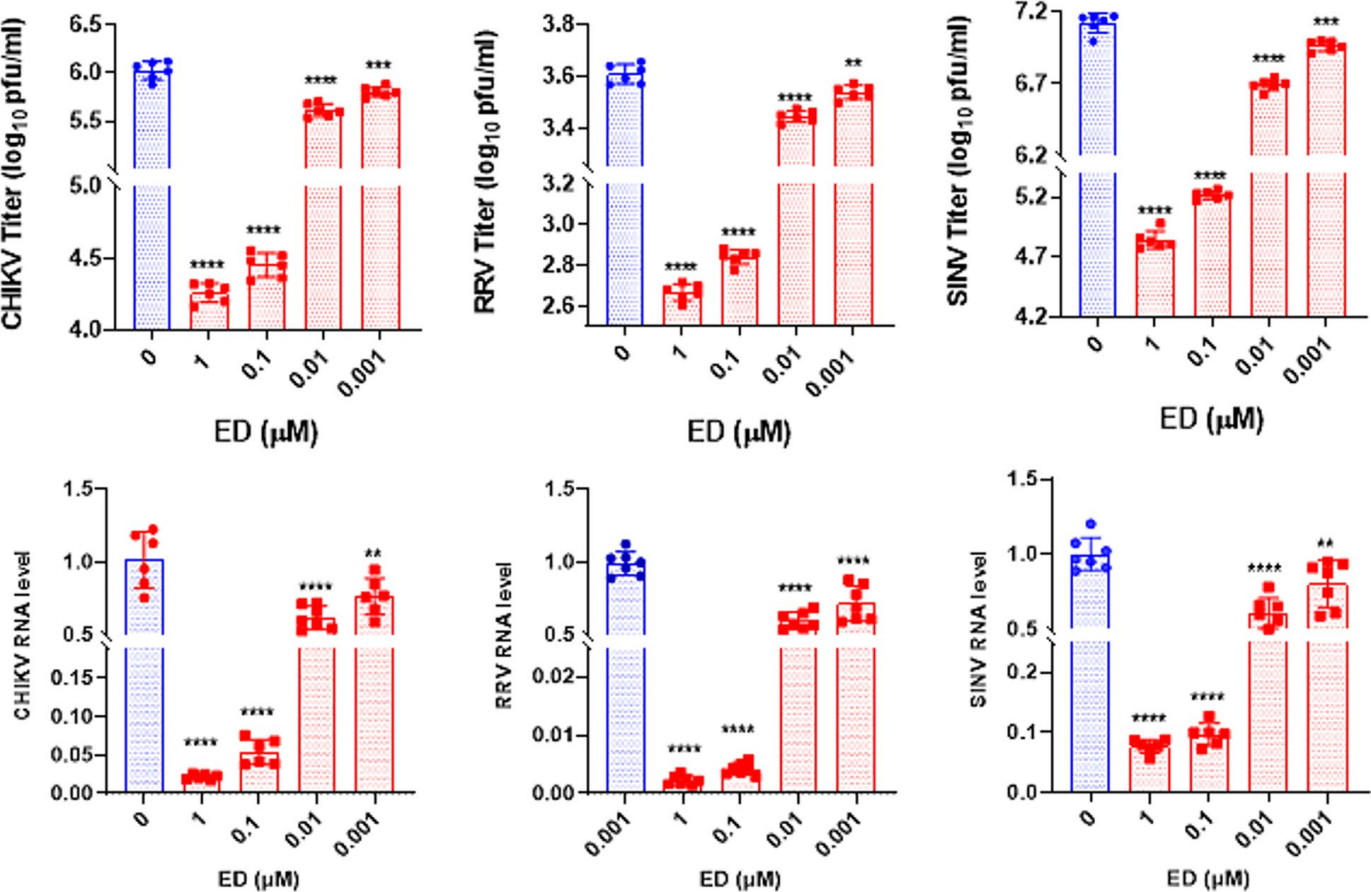
ED inhibits the replication of SINV and RRV. ERMS cells were infected with CHIKV, RRV or SINV (1 MOI) and incubated with different concentrations of ED. The cells and culture supernatants were harvested at 6 h pi for RNA extraction and viral titer determination, respectively. The viral titers determined by plaque assay are shown in the top panel. The relative viral RNA levels are shown in the bottom panel, where *Gapdh* was used as the internal control. Data represent mean ± SD from two independent experiments, each performed in triplicate

The nsP2 amino acid sequences of the alphaviruses RRV and SINV showed about 69% and 58% sequence similarity to the nsP2 protein of CHIKV (Fig. S3). Out of the nine amino acids of the nsP2 helicase that lined ED within the 4.0 Å space in our in silico studies, eight were conserved in RRV and seven in SINV (Fig. S3). With a view to examine the pan-alphavirus antiviral potential of ED, we studied the replication of these two viruses in the presence of ED. ED significantly reduced viral RNA and titers in RRV- or SINV-infected ERMS cells at concentrations of 0.1 µM and as low as 0.001 µM (Fig. 13) indicating its broad anti-alphavirus activity.

## Discussion

Several antiviral molecules have been identified against CHIKV using in vitro and in silico methods. However, these are still in the experimental phase and have not been taken through the drug development pathway, which is long, expensive, and tedious. Drug repurposing, or repositioning, is the process of finding new therapeutic uses for existing drugs that were originally developed for different indications. This approach is particularly advantageous for antiviral drug development because it leverages known safety profiles, manufacturing data, and pharmacokinetics of approved or investigational drugs, reducing the time and cost involved in bringing a new antiviral therapy to market. We screened the Spectrum Collection of 2560 compounds that includes all the compounds in the US and international drug collections and have identified ED as a potent antiviral molecule against CHIKV in cell culture and a mouse model of disease. The molecule with an IC_50_ of 10 nM showed a high selectivity index (SI) of 967, demonstrating its high potential for further development as a CHIKV antiviral (Fig. 1B).

ED is the dichloride salt of emetine, a natural alkaloid derived from the roots of *Psychotria ipecacuanha* (ipecac), recognised for its emetic and antiprotozoal properties [59]. Emetine has a long history of use, primarily as an antiemetic to induce vomiting, and as an antiprotozoal drug against *Entamoeba hystolytica* [60], until a safer metronidazole became available. The emetine-induced non-permanent cardiotoxicity was unequivocally associated with the high-dose (60 mg/day for 10 days) required to achieve a minimum inhibitory concentration of 25 µM against *Entamoeba hystolytica*. However, toxicity was more commonly observed when treatment exceeded 5 days. No cardiovascular side effects were reported when emetine was used for various indications at a low dose of 20 mg/day [54].

In the past, emetine has been used to treat the Spanish influenza [54], the shingles [61, 62], adenoviral kerato-conjunctivitis [63], and hepatitis [64, 65]. In recent years, emetine has garnered attention as a potential therapeutic agent for viral infections, showing potent antiviral activity at IC_50_ values in the sub-micromolar or low nanomolar ranges [54]. A phase 2/3 trial is ongoing to determine emetine's efficacy and safety for the treatment of symptomatic COVID-19 in a randomised clinical trial (ClinicalTrials.gov ID NCT05889793) at the Johns Hopkins University, USA.

Emetine has demonstrated antiviral potential against a wide range of viruses, including RNA and DNA viruses. For example, it has been shown to inhibit the replication of the RNA genome-containing Zika virus (ZIKV), Ebola virus (EBOV) [66], Echovirus 1 human metapneumovirus, Rift Valley fever virus [67], coronaviruses such as hCoV-NL63, hCoV-OC43, MERS-CoV [68], SARS-CoV-2 [47, 69–72], human enteroviruses including EV-A71, CV-A16, CV-B1, EV-D68, Echov-6 [53] and foot-and-mouth disease virus (FMDV) [72]. Emetine also inhibited DNA viruses, such as Herpes simplex virus type 2 [67] and Human cytomegalovirus (HCMV) [73]. The efficacy of emetine has also been demonstrated in preclinical studies in animal models against HCMV [73], ZIKV [66], and Enterovirus-A71 [53].

CHIKV replication in skeletal muscle cells is required for disease development, and replication in skeletal muscle cells is a critical mediator of CHIKV pathogenesis [74]. Mounting evidence has implicated skeletal muscle as an important site of CHIKV disease development [75–79]. Therefore, in addition to the commonly used BHK-21 cells, we studied ED-mediated CHIKV inhibition in ERMS cells. These cells of human origin resemble undifferentiated muscle cells (myoblasts), a physiologically relevant target of CHIKV infection. ERMS cells retain characteristics of the skeletal muscle lineage, including expression of muscle-specific markers such as myogenin and desmin [79], thereby providing a biologically relevant system to investigate ED’s antiviral effects in a cell type associated with CHIKV-induced musculoskeletal pathology.

ED inhibited CHIKV replication in ERMS cells in the nanomolar range with an IC_50_ of 10 nM, and a complete inhibition of virus replication was seen at a 50 nM concentration. Importantly, in a mouse model of CHIKV infection, ED showed robust antiviral properties, where ED-treated CHIKV-infected mice showed lower viremia and did not develop characteristic footpad edema (Fig. 2). The weight loss associated with CHIKV infection was also reversed in the ED-treated animals. A clear dose–effect was observed on clinical symptoms of viremia and footpad edema in CHIKV-infected mice treated with graded doses of ED (Fig. 3). Mice that were treated with ED as late as 48 h after CHIKV exposure showed significantly reduced viremia and absence of clinical symptoms of footpad edema (Fig. 4).

While ED targets RNA replication early during infection, this ED effect would continue to suppress RNA replication even at later time points, reducing the virus titers. Consistent with this, our in vitro data demonstrated strong antiviral activity of ED when administered after infection, resulting in rapid inhibition of viral RNA accumulation (Fig. 6). At 48 h pi, in the CHIKV-infected mice, different tissues and cells may have the virus replicating at different stages of virus life cycle. In the 48 h treatment group, blood samples were collected 3–4 h after the drug treatment. CHIKV replicates rapidly showing ~ tenfold titer increase per cycle in Vero/muscle cells [80]. The time window of 3–4 h will allow at least one replication cycle to be inhibited, resulting in reduced RNA levels and virus titers in blood.

Footpad edema in mice is induced by CHIKV replication and associated immunopathology [81]. Several observations support antiviral activity as an important driver of edema reduction in mice in the present study: (i) ED reduces circulating viral RNA and infectious virus titers in vivo, (ii) time-of-addition and replication kinetics experiments demonstrate a virus replication-inhibition mechanism, and (iii) nsP2-targeted mechanistic data provide a defined molecular basis for viral inhibition. While direct biochemical confirmation of nsP2 helicase inhibition in vivo is technically challenging, together, these findings support that reduced viral replication is an important explanation for the improved disease outcome in mice.

Importantly, ED was effective in mice at a SC dose of 6 mg/kg/d, which translates to a 0.49 mg/kg/day human dose. This is significantly lower than the 1 mg/kg/day dose used in humans (60 mg/day) to treat amoebiasis, which was causing some non-permanent adverse effects [54]. It may be noted that no cardiovascular side effects were reported when emetine was used for different indications at a low dose of 20 mg/day [54].

The ED-mediated inhibition of CHIKV replication, as studied by the kinetics of viral RNA synthesis and the extracellular virus titers in the infected cells, indicated that ED inhibited the early stage/s of CHIKV replication (Fig. 5). The time-of-drug-addition studies also suggested that ED affected early events during the CHIKV life cycle (Fig. 6). During alphavirus replication, the incoming positive-strand RNA is first translated to produce non-structural proteins, after which de novo RNA synthesis begins with negative-strand formation. The nsP2 helicase plays a critical role in RNA replication by unwinding double-stranded RNA (dsRNA) intermediates formed during replication [82, 83]. Therefore, inhibition of nsP2 helicase activity would impair early negative-strand RNA synthesis, followed by reduced synthesis of positive-strand genomic RNA. ED treatment caused an early, dose-dependent reduction in negative-strand RNA levels, followed by decreased positive-strand genomic RNA levels, consistent with inhibition of nsP2 helicase–dependent replication complex activity and impaired CHIKV RNA synthesis.

The significantly reduced CHIKV RNA levels in virus-infected cells as early as 3 h pi indicated an inhibition of the virus binding or uptake step. While virus binding was not affected, CHIKV uptake was reduced in ERMS cells in the presence of ED concentration as low as 0.1 µM. ED has previously been reported to inhibit the entry of Ebola pseudovirus into HeLa cells [66], Hantaan virus into VeroE6 cells [84] SARS-CoV-2 into Vero cells [47], and Enterovirus-A71 into rhabdomyosarcoma cells [53], although the underlying mechanisms were not investigated.

We ruled out any virucidal effect that ED might have on CHIKV virions by directly incubating the virus with ED. Emetine has been reported to inhibit viral entry by disrupting host lysosomal function [66]. A previous in silico molecular docking study identified CHIKV nsP2 helicase and envelope glycoprotein as potential viral targets of emetine [56]. Therefore, the inhibition of CHIKV uptake observed at 0.1 μM ED may be attributable to interference with viral entry processes, possibly through interactions with the viral envelope glycoprotein and/or disruption of host lysosomal function. While no significant inhibition of host protein synthesis was seen at 0.1 µM ED concentration, even a small inhibition could affect endocytosis, membrane trafficking, cytoskeletal dynamics, receptor recycling, or virus–cell interactions. This could result in lower virus uptake and thereby reduced virus titers. While the role of reduced virus uptake in ED’s antiviral activity cannot be ruled out, ED’s robust antiviral activity when added post-entry in the time-of-addition experiment argues against entry inhibition being the dominant mechanism.

The multifunctional nsP2 is one of the most important CHIKV non-structural proteins, which has a protease domain containing the activity required for producing the mature non-structural proteins, and a helicase domain essential for unwinding RNA to facilitate the viral RNA replication and transcription. Our data showed that ED strongly inhibited replication of CHIKV RNA. Consistent with a previous study [56], our in silico studies predicted ED binding to the helicase domain of the nsP2 protein with high affinity (Table 1, Fig. 10). This observation was experimentally validated using the ITC and MST methods, confirming the binding of ED with the helicase domain of nsP2 with a *K*_d_ value of 2.56 µM and 3.06 µM, respectively (Fig. 8). Furthermore, a mutation of the in silico-predicted ED-interacting nsP2 residue caused a significant reduction in the binding affinity of nsP2 with ED; the *K*_d_ value for the mutant nsP2 was 29.40 µM (Fig. 10E). Importantly, ED binding to the nsP2 protein inhibited its helicase activity in vitro in a dose-dependent manner (Fig. 9B). The MST studies showed that ED bound in or around the RNA binding site of the nsP2 helicase (Fig. 11). Our data thus indicate that ED may suppress CHIKV replication by binding to its nsP2 protein and inhibiting its helicase activity. Since we have not studied other CHIKV proteins, their role in the ED-mediated anti-CHIKV activity cannot be ruled out, even though in silico studies did not predict their interaction with ED.

Emetine has been shown to exhibit its antiviral effects primarily by interfering with viral replication processes. Interestingly, it uses a variety of mechanisms for its antiviral action. Emetine is a well-characterized inhibitor of eukaryotic protein synthesis via targeting the 40S ribosomal subunit [85]. This may also prevent the translation of viral proteins, contributing to emetine’s antiviral action. For enterovirus EV-A71, emetine suppressed replication by inhibiting viral IRES-driven translation [53]. Emetine’s antiviral activity against ZIKV and EBOV involves its inhibition of ZIKV RNA-dependent RNA polymerase activity to block viral replication and disruption of lysosomal function to impair EBOV entry in cell culture [66]. Against SARS-CoV-2, emetine acts by blocking the interaction between viral mRNA and the host eukaryotic initiation factor 4E (eIF4E), thereby suppressing viral RNA and protein synthesis without significantly affecting viral attachment or entry [86]. Emetine showed virucidal activity as well as inhibited the replicase activity of FMDV [72]. In the case of MERS-CoV, emetine blocked the virus entry in a pseudovirus entry assay [68]. In the case of HCMV, a DNA virus, emetine inhibits virus replication by inducing nuclear translocation of ribosomal protein S14 (RPS14), which binds MDM2 and disrupts HCMV-induced interactions between MDM2-p53 and MDM2-viral IE2, thereby blocking early viral gene expression after entry but before DNA replication [73]. Here, we have shown yet another virus-directed antiviral mechanism of emetine. ED was shown to bind CHIKV nsP2 and inhibit its helicase activity, resulting in reduced viral RNA synthesis and suppressed viral titers.

The low IC_50_ of 10 nM and the complete inhibition of CHIKV replication at 50 nM ED suggest that ED-mediated protein synthesis inhibition may not have a significant role in the ED-mediated CHIKV inhibition in ERMS cells, since host cell protein synthesis was largely unaffected at 100 nM ED concentration as seen using puromycin labelling (Fig. 7B). Importantly, viral nsP2 protein levels were markedly reduced at concentrations where host protein translation remained largely intact (Fig. 7A) supporting a virus-specific mechanism rather than generalized protein synthesis inhibition. Second, the time-of-addition experiment showed maximal inhibition during the post-entry replication phase, consistent with interference in viral RNA replication and non-structural protein synthesis. A generalized protein synthesis inhibition mechanism would be expected to produce comparable virus inhibition regardless of the timing of addition, which was not observed. It should be noted that the puromycin-labelling method is qualitative or at best semi-quantitative, and even a small inhibition of translation could significantly reduce viral replication, especially during the early stages of the virus life cycle. Thus, while contributions from host translational modulation cannot be excluded, nsP2-mediated selective inhibition of CHIKV replication appears to be a promising process contributing to ED’s antiviral activity. Polysome profiling of cells in the presence of graded doses of ED would be a more sensitive method to address the role, if any, of ED-mediated host cell protein synthesis inhibition in CHIKV replication inhibition.

The N-terminal domain of CHIKV nsP2 contains both the helicase and ATPase activity [40, 41]. In our study, ED inhibited the helicase activity without affecting the ATPase activity (Fig. 9B). This functional selectivity could be due to the spatial separation of nsP2’s helicase and ATPase catalytic sites [33, 40]. CHIKV nsP2’s ATP-binding pocket lies within the cleft between the RecA1 and RecA2 domains, connected through a connector to form the ATP entry tunnel. Trp450 residue in the connector forms a stacking interaction with the ADP adenine base. Key catalytic residues present in the ATP active site include Lys192/Ser193 (Motif I), Asn213 (Motif Ia), Asp252/Glu253 (Motif II), Gln283 (Motif III), Arg311/Arg312 (Motif IIIa), and Arg416 (Motif VI). In contrast, the RNA-binding site, where we have shown ED binding, forms a central groove atop the RecA1/RecA2 domains, partially covered by the NTD-stalk-1B region. The ssRNA interacts with the conserved RNA backbone recognition motifs present on the top of the two helicase core domains (Lys211, Thr233, Asp235, Ser236, Trp363, Arg361, His409, etc.), while the most unique stacking interactions between the nsP2 helicase and the ssRNAare mediated by Tyr161 and Phe164 in the N-terminal domain, and Phe287 in RecA1, required for the unwinding [33]. ED binds around this RNA-interaction region (~ 25–35 Å below the RNA-binding groove), thus disrupting RNA binding without affecting ATPase activity. Consistent with this, mutations in the RNA binding region (e.g., Phe164, Tyr161) were shown to impair RNA stacking and unwinding but not affect ATPase activity [33], highlighting the functional independence of these processes. It may be noted that residues Tyr161, Phe164, Phe287, Lys211, T233, His 409, and Asp235 of CHIKV nsP2 were identified in our study as being involved in ED binding (Table S3). These residues were previously shown to be important for helicase activity [33]. Thus, ED acts as a selective CHIKV nsP2 helicase inhibitor by disrupting RNA-binding interactions, while sparing ATPase activity.

In our in silico and in vitro studies, ED was shown to bind to the helicase domain of the multifunctional CHIKV nsP2 protein. Resistance mapping through virus passage under drug pressure is a standard approach to validate the drug target. Drug resistance selection through virus passaging is an unpredictable empirical method and often fails to isolate the mutant even after prolonged passaging [87–89]. A previous report showed that emetine inhibited replication of diverse RNA and DNA viruses without generating drug-resistant variants even after 25 serial passages [90]. We, therefore, produced an nsP2 helicase mutant by site-directed mutagenesis and demonstrated its compromised ED binding (Fig. 10E), validating the ED target. Nonetheless, resistance mapping would further strengthen the causal linkage of ED binding to CHIKV nsP2.

Targeting the nsP2 helicase has broad biological and therapeutic implications because this enzyme sits at the core of alphavirus replication and is both functionally essential and evolutionarily conserved. The nsP2 protein is a multifunctional component of the viral replicase complex, with its N-terminal helicase/NTPase activity required for unwinding RNA intermediates and enabling efficient genome replication, making it indispensable for viral replication [82, 91]. Importantly, nsP2 helicase sequence and structural motifs are highly conserved across alphaviruses including CHIKV, SINV, and RRV (Fig. S3), and therefore inhibitors of this domain have high potential as broad-spectrum (pan-alphavirus) antivirals [92]. Indeed, in our studies, ED was shown to inhibit CHIKV, RRV, and SINV, the three alphaviruses that were tested. Despite the demonstrated sequence and structural conservation in the nsP2 helicase, role of ED in inhibiting SINV and RRV helicase remains to be shown. In a recent study, oxaspiropiperidine allosteric inhibitors of nsP2 demonstrated broad activity against CHIKV, and Venezuelan equine encephalitis viruses, underscoring the potential of this enzyme as a target for pan-alphavirus therapy [93]. In addition to its direct role in RNA synthesis, nsP2 also regulates host–virus interactions, including suppression of host antiviral responses [82]. Therefore, pharmacological inhibition of nsP2 helicase activity is expected to disrupt multiple stages of the viral life cycle simultaneously, RNA replication, replicase complex formation and function, and host response evasion, thereby reducing the likelihood of drug resistance development. Collectively, these findings position nsP2 helicase as a central, druggable target for the development of broad-spectrum antiviral strategies against alphaviruses.

The arthritogenic symptoms observed during the acute phase of CHIKV infection are largely due to the host's pro-inflammatory responses, with elevated levels of cytokines and chemokines playing pivotal roles in the development of joint inflammation and pain [7, 94]. Emetine's anti-inflammatory effects suppress pro-inflammatory cytokines TNFα, IL-1β, and IL-6 via potent NF-κB pathway inhibition through IκBα phosphorylation blockade [95–97]. This mechanism could effectively counter the cytokine storm characteristic of severe CHIKV infection, where elevated plasma levels of IL-1β and IL-6 alongside decreased RANTES strongly correlate with disease severity, persistent high fever, and debilitating joint pathology, including arthralgia [98]. Since CHIKV-induced joint swelling is driven by inflammatory responses, the observed reduction in the mouse footpad edema may partially reflect anti-inflammatory rather than purely antiviral activity. Therefore, the role of NF-κB-mediated ED action in controlling CHIKV replication in the cells, and particularly in the animal model, needs further investigation. This could be addressed by conducting histopathological studies and measuring inflammatory cytokines in treated animals.

Interestingly, ED is more potent in antiviral assays (IC_50_ ≈ 10 nM) than its direct binding to nsP2 (*K*_d_ ≈ 3 µM) would suggest. This is not an uncommon phenomenon in drug discovery as they measure fundamentally different properties: IC_50_ reflects functional potency in a specific in vivo assay, while *K*_d_ measures intrinsic binding affinity at equilibrium in vitro [99]. Thus, significant differences between cellular antiviral activity inhibition and NS3 helicase activity inhibition in vitro were reported for Yellow Fever [100], Japanese encephalitis, and hepatitis C viruses [101]. This gap in the *K*_d_ and IC_50_ values suggests that the ED’s effectiveness in a living system is influenced by more than just its "grip" on the target protein. Thus, while ED binding to CHIKV nsP2 helicase may be a promising mechanism involved in ED’s antiviral action, the role of other mechanisms, such as reduced virus uptake or anti-inflammatory properties of ED cannot be disregarded.

Acute CHIKV infection often leads to a debilitating chronic phase, with persistent musculoskeletal pathology affecting up to 60% of patients for months or years; the primary driver is viral RNA and protein persistence within synovial macrophages, triggering a sustained inflammatory response [102]. The mouse model of CHIKV infection used in our studies is limited to short‑term, acute endpoints, including viremia, paw edema, and body‑weight changes. Non-human primate and C57BL/6 mouse models exist for studying post-acute or long-term CHIKV outcomes beyond short-term acute endpoints, although no single model fully replicates all human chronic features [103, 104]. It is thus important to study the usefulness of ED in controlling the long-term sequelae of CHIKV infection in an appropriate animal model.

Overall, ED likely exerts antiviral activity through a combination of mechanisms, with nsP2 helicase inhibition representing a promising virus-directed mechanism, while reduced virus uptake and host-directed effects may also contribute to the antiviral phenotype. The robust anti-CHIKV activity in cell culture and the therapeutic action of ED in the animal model, especially at a dose smaller than the prescribed human dose, indicate its significant antiviral potential. Additionally, emetine’s anti-inflammatory properties may mitigate the cytokine storm associated with CHIKV infection [105]. An emetine concentration of 25 µM is required to inhibit *Entamoeba histolytica* growth in vitro [106], whereas near-complete inhibition of CHIKV replication was seen at a concentration of 50 nM in the cell culture. These concentrations are around 500-fold lower than the emetine dose used to kill the amoeba. Previously published pharmacokinetic studies indicate that emetine achieves sustained tissue exposure and concentrations exceeding the reported antiviral EC₅₀ values at tolerable doses in mice [47, 73]. These data suggest that ED’s antiviral efficacy observed in vivo is achievable within pharmacologically relevant exposure ranges, although detailed understanding of ED’s antiviral mechanism, its comprehensive safety and PK/PD evaluation will be required to determine the feasibility of repurposing ED as a viable CHIKV antiviral agent.

## Conclusion

Library screening for drug repurposing, which accelerates antiviral development by leveraging existing drugs' established safety profiles, identified ED as a potent CHIKV inhibitor in cellular and animal models. In silico and in vitro studies revealed that ED targets the CHIKV nsP2 helicase, disrupting viral replication. While these findings suggest ED as a promising CHIKV antiviral, further investigation is needed to fully understand its mechanism(s) before advancing toward therapeutic application.

## Non-technical summary

Chikungunya virus (CHIKV) spreads through the bites of virus-infected *Aedes* mosquitoes. The virus causes frequent epidemics of chikungunya fever involving severe joint edema and muscle pain. Viral activity has been recorded in more than 100 countries in Asia, Africa, South and Central America, and Europe, and it has become endemic in several regions of the world. In 2024, around 480,000 CHIKV cases and over 200 deaths were reported worldwide. While a CHIKV vaccine was recently approved, no virus-specific antiviral therapy is available. Considering the global medical significance of the virus, the development of effective CHIKV antivirals is a priority. With a view to repurposing an existing drug, we screened a collection of all approved US and international drug molecules and identified emetine dihydrochloride (ED) as a potent inhibitor of CHIKV replication in a cell culture model. CHIKV-infected mice treated with ED showed reduced virus replication and no joint swelling, which is a typical clinical symptom of CHIKV disease. Our studies show that ED binds CHIKV nsP2 protein, inhibiting its helicase activity, which is required for replicating the viral genomic RNA and producing the viral proteins. In the past, emetine has been used in humans to treat amoebiasis. While CHIKV helicase inhibition represents a promising antiviral mechanism, further investigation of ED's host-directed actions is warranted. A complete mechanistic profile is prerequisite to emetine's therapeutic repurposing for CHIKV patients.

## Supplementary Information

Supplementary material 1.

## Data Availability

No datasets were generated or analysed during the current study.
