# Supplementary material for: Emetine dihydrochloride inhibits Chikungunya virus nsP2 helicase and shows robust antiviral activity in cells and mice
Source: J Biomed Sci. 2026 Jul 20;33:75. doi: 10.1186/s12929-026-01280-9 (PMC13386891; doi:10.1186/s12929-026-01280-9)
Supplement: Supplementary file 1 — Supplementary material 1. [file 12929_2026_1280_MOESM1_ESM.pdf]

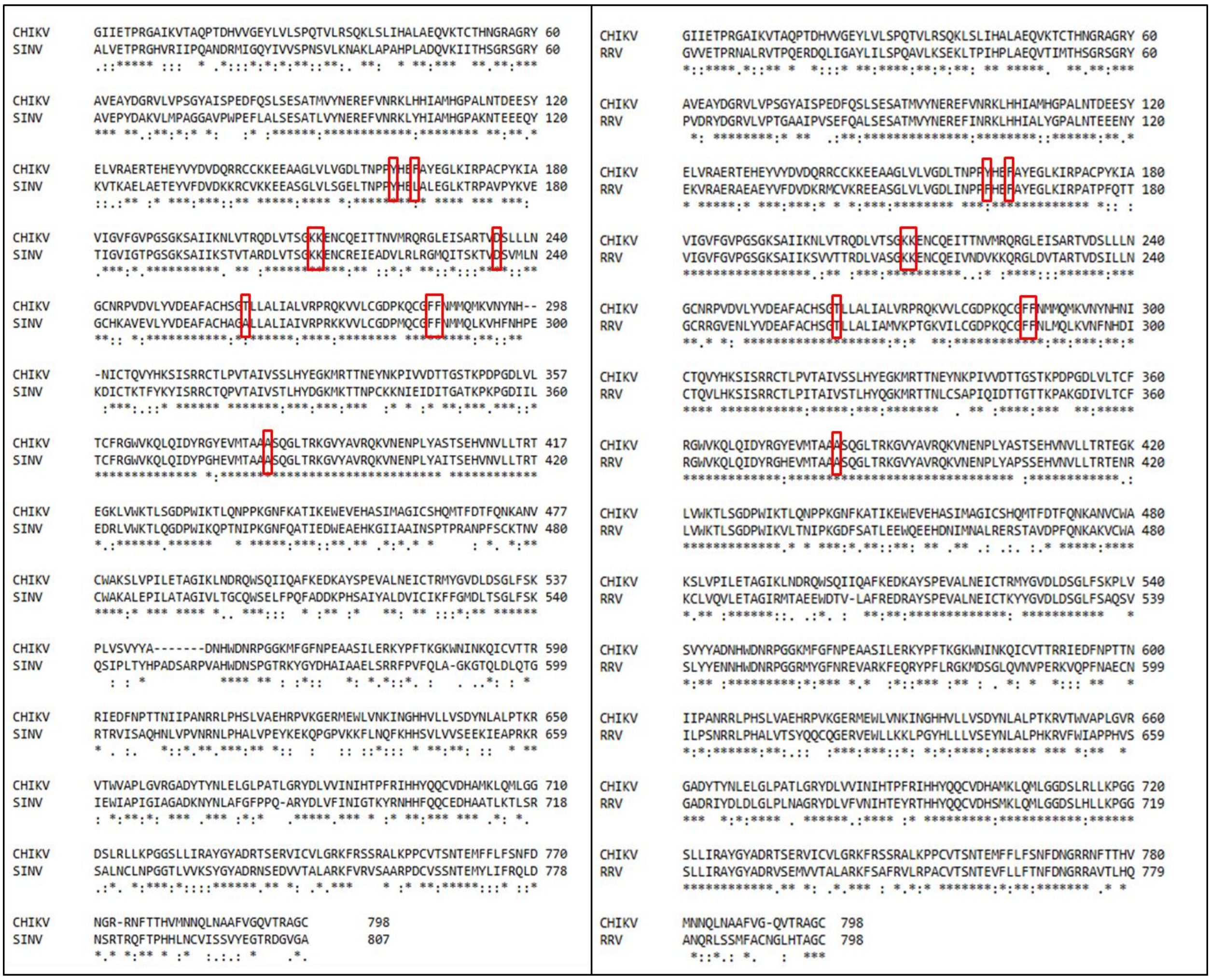

Figure S1. Conservation of the nsP2 sequence among CHIKV, SINV and RRV.

The amino acid sequence of the CHIKV nsP2 protein was aligned with the SINV and RRV nsP2 proteins using the UniProt Align tool. The amino acids lining within the 4.0 Å space of the nsP2 interaction with ED, as identified *in silico*, are shown in red coloured boxes.

Table S1

Table S1: Anti-CHIKV activity of the 164 compounds at 1 µM concentration in different cell lines.

S. No.	Name of the compound	ERMS		BHK-21		Vero	
		% CHIKV inhibition	% Cell viability	% CHIKV inhibition	% Cell viability	% CHIKV inhibition	% Cell viability
1	Sanguinarium chloride	96.71	81.51	18.78	89.66	20.43	91.28
2	Amodiaquine dihydrochloride	16.47	104.57	0.45	93.65	2.48	101.73
3	Benzethonium chloride	29.56	108.71	8.49	94.61	9.39	99.21
4	Bithionol	3.20	106.90	-14.09	95.09	10.92	103.57
5	Cetylpyridinium chloride	41.20	98.65	4.70	97.71	18.69	102.10
6	Chlorhexidine dihydrochloride	-14.18	98.51	2.43	95.10	12.93	97.54
7	Chlorpromazine hydrochloride	1.24	104.48	-13.81	91.79	1.63	99.74
8	Clomiphene citrate	13.30	102.80	-22.76	94.54	4.55	103.36
9	Clotrimazole	6.96	94.05	-4.67	100.60	7.26	100.23
10	Bazedoxifene acetate	-5.56	90.91	-14.87	89.66	-3.04	98.83
11	Daunorubicin hydrochloride	12.15	90.92	68.88	78.97	-4.32	93.36
12	Pyrithione zinc	97.72	98.58	99.91	81.49	96.62	89.57
13	Disulfiram	87.22	106.38	-10.23	99.51	0.87	116.07
14	Emetine dihydrochloride	98.36	98.55	100.00	86.34	100.00	86.40
15	Estradiol cypionate	-13.75	118.89	5.83	100.96	1.86	107.30
16	Doramectin	-16.62	118.35	-66.07	98.28	-2.65	105.58
17	Gentian violet	98.62	93.96	90.23	44.99	81.17	86.44
18	Gramicidin A	98.83	82.07	35.77	85.27	43.25	111.75
19	Hexachlorophene	52.42	109.33	-6.74	97.70	39.98	109.69
20	Robenidine hydrochloride	-8.01	104.32	-4.61	95.24	0.49	110.41
21	Miconazole nitrate	2.26	120.35	-2.84	112.21	20.02	120.20
22	Prochlorperazine edisylate	16.14	125.96	9.90	110.93	3.94	112.44
23	Pyrimethamine	-10.53	119.18	7.04	89.15	2.88	110.60
24	Tamoxifen citrate	2.39	121.41	24.98	103.36	-4.19	115.39
25	Thimerosal	98.33	94.90	20.44	93.55	90.80	101.23
26	Thioridazine hydrochloride	5.68	120.87	8.15	105.80	2.31	110.07
27	Triamterene	-5.93	120.85	20.98	96.45	-3.06	114.67
28	Trifluoperazine hydrochloride	2.11	114.74	8.33	110.92	-2.29	121.43
29	Phenylmercuric acetate	98.43	88.23	16	105.13	89.16	85.25
30	Aminacrine	25.53	101.41	0.66	98.46	8.63	112.79
31	Fluphenazine hydrochloride	1.56	118.85	8.76	102.20	-1.69	110.61

32	Puromycin dihydrochloride	95.43	110.63	37.69	103.85	2.25	114.15
33	Pimozide	35.75	121.03	26.60	106.18	-5.61	117.67
34	Sulconazole nitrate	17.52	117.35	24.94	109.17	10.32	117.08
35	Suloctidil	71.74	127.84	-15.03	107.51	83.99	131.59
36	Econazole nitrate	21.75	114.72	19.47	109.81	26.86	117.25
37	Raloxifene hydrochloride	15.25	116.66	-0.33	98.63	20.62	110.38
38	Cycloheximide	98.14	107.08	99.88	91.15	99.99	118.32
39	Monensin sodium	96.10	112.19	-10.71	102.90	8.69	109.68
40	Isoconazole nitrate	1.40	104.76	-5.62	110.67	10.14	122.10
41	Adapalene	-3.83	113.45	-10.44	95.29	-4.55	101.57
42	Perphenazine	15.33	122.62	1.94	110.00	-0.73	114.26
43	Mefloquine hydrochloride	3.79	124.80	2.69	109.00	2.05	116.15
44	Chlorprothixene hydrochloride	18.89	126.72	14.92	111.20	2.67	112.67
45	Perhexiline maleate	60.23	128.31	4.59	116.26	12.45	116.30
46	Lapatinib	42.05	124.79	-55.36	119.99	15.71	112.37
47	Methylbenzethonium chloride	21.79	112.24	17.99	113.55	4.26	109.31
48	Amsacrine	21.85	90.78	55.99	86.77	-9.21	82.55
49	Cetalkonium chloride	4.72	112.31	11.30	113.88	4.55	106.48
50	Naltrexone hydrochloride	-0.10	103.93	8.10	102.94	10.14	108.89
51	Niclosamide	98.19	100.22	88.0	105.09	89.77	107.24
52	Mitoxantrone hydrochloride	80.95	94.62	56.98	83.76	-3.81	83.14
53	Thiram	95.65	100.74	-16.32	107.87	3.76	115.51
54	Penfluridol	94.75	112.40	24.23	106.11	9.93	112.31
55	Benzalkonium chloride hydrate	19.64	119.59	27.64	106.45	1.17	109.58
56	Terfenadine	-1.69	115.83	21.10	107.87	-3.42	110.39
57	Selamectin	5.45	113.79	-18.84	103.59	17.91	112.23
58	Triflupromazine hydrochloride	-1.23	110.49	7.98	113.09	0.38	109.89
59	Nitazoxanide	12.81	105.96	-13.63	108.30	17.97	111.15
60	Candicidin	-2.60	103.17	1.27	106.91	-1.49	103.31
61	Avobenzone	0.69	110.02	7.30	106.79	1.39	110.85
62	Closantel	-9.03	115.17	6.59	110.67	-0.18	103.38
63	Amlodipine besylate	11.58	126.09	-27.23	119.57	13.70	127.32
64	Apramycin sulfate	-7.53	116.63	12.83	110.20	2.45	108.09
65	Sertraline hydrochloride	52.26	117.16	-8.15	106.26	2.13	106.56

66	Nonoxynol-9	4.71	111.35	-6.21	110.76	3.41	106.87
67	Bifonazole	13.82	104.05	-1.62	108.42	11.40	103.30
68	Moxidectin	-0.16	103.39	6.33	107.28	2.69	104.79
69	Fluorescein	91.34	98.91	42.17	104.43	-76.55	102.35
70	Rafoxanide	-8.93	93.63	8.42	113.01	4.76	101.95
71	Lynestrenol	-0.19	97.41	-28.58	99.87	3.42	98.35
72	Triclosan	10.64	104.13	-5.05	98.54	7.87	100.19
73	Chlorothalonil	97.05	82.35	71.26	78.02	98.36	49.86
74	Bepridil hydrochloride	26.25	103.51	-13.38	102.88	7.78	101.44
75	Clofazimine	39.60	98.13	-19.00	88.47	19.59	98.68
76	Amoxapine	-2.79	100.27	-22.44	98.76	2.71	98.95
77	Toremifene citrate	1.20	96.98	-22.71	100.38	6.16	94.74
78	Vortioxetine hydrobromide	21.45	103.81	0.55	105.63	11.79	111.55
79	Amiodarone hydrochloride	14.49	103.90	-9.87	99.79	14.19	126.13
80	Evans blue	29.64	106.95	30.50	98.58	8.41	110.89
81	Ivermectin	2.99	108.30	-7.79	98.66	10.02	121.47
82	Mitotane	13.37	109.23	-13.19	98.45	21.92	129.51
83	Asenapine maleate	2.96	109.18	2.71	95.99	28.62	119.78
84	Quinestrol	13.93	109.94	-27.18	97.95	15.07	113.86
85	Desogestrel	1.35	117.27	-6.44	103.91	19.63	110.17
86	Ebastine	33.79	111.60	-8.95	99.82	12.08	116.56
87	Abamectin	26.84	107.47	6.28	98.78	28.00	103.63
88	Gemcitabine hydrochloride	68.90	101.19	24.66	77.15	56.50	72.98
89	Oxyclozanide	0.20	114.79	5.92	106.90	6.60	107.54
90	Benzyl alcohol	-5.91	119.58	5.62	106.84	15.81	83.33
91	Acriflavinium hydrochloride	-265.37	112.96	-15.00	104.12	-46.05	94.59
92	Broxyquinoline	10.04	115.10	14.30	104.66	11.01	118.63
93	Dichlorophen	5.16	119.75	0.77	106.02	8.33	94.82
94	Hexetidine	10.23	118.08	-7.00	106.73	12.24	116.08
95	Bronopol	17.34	118.75	16.78	104.25	12.69	125.07
96	Clofoctol	26.11	120.78	-8.96	108.94	20.37	121.89
97	Benzyl isothiocyanate	58.23	111.95	11.27	109.45	0.94	109.72
98	Salinomycin	95.04	109.33	-10.33	108.86	52.50	113.36
99	Broxaldine	40.23	95.80	7.37	96.95	5.23	99.48
100	Escin	52.20	120.91	7.50	116.76	10.13	102.99

101	Proflavine hemisulfate	-251.99	120.65	14.66	107.99	-36.03	99.91
102	Benzylhydrazine hydrochloride	7.89	121.81	12.08	113.81	-2.07	101.86
103	Naringenin	11.09	125.10	21.52	106.87	2.40	94.52
104	Agmatine sulfate	2.50	125.75	19.16	115.57	-31.66	41.47
105	Dipicolinic acid	0.90	123.52	24.74	109.52	2.64	102.80
106	3,4'-dimethoxyflavone	13.19	118.56	23.21	109.27	8.32	97.66
107	3,5-dihydroxyflavone	6.01	120.92	24.51	115.39	2.75	104.28
108	Patulin	89.84	108.63	60.49	89.85	96.0	80.1
109	Artepaulin	-13.85	116.34	20.39	111.34	3.99	100.02
110	Dihydrorotenone	52.56	91.61	21.11	100.58	32.22	86.91
111	Allopregnanolone	25.12	122.70	-0.23	105.55	-0.56	97.02
112	Galangin 3-o-methyl ether	-11.74	130.11	22.03	109.18	-2.82	88.14
113	6,7-dihydroxyflavone	0.49	128.49	20.52	109.52	5.58	113.72
114	Parthenolide	37.20	118.39	6.12	113.32	-0.64	93.98
115	Methyl gamboginate	34.24	122.48	21.34	109.34	1.52	114.56
116	Anhydrobrazilic acid	14.54	123.80	18.81	110.05	-4.24	91.95
117	16-EPOXY-21-aco-5-PREGNENOLONE	22.65	126.02	14.37	111.03	0.38	109.86
118	AVERMECTIN a1a	-8.70	119.52	2.10	111.09	0.00	126.12
119	Cantharidin	6.47	125.34	2.58	100.40	-1.97	104.01
120	Anthothecol	83.00	99.0	76.64	90.69	41.51	78.90
121	Obtusaquinone	65.94	99.11	14.62	90.25	81.00	77.91
122	Gambogic acid	97.19	112.35	99.95	81.03	100.00	71.30
123	CHOLESTAN-3b,5a,6b-TRIOL	47.74	125.46	-12.18	108.65	1.29	89.49
124	Piplartine	21.33	113.15	20.06	108.66	2.27	115.33
125	Plumbagin	73.38	107.95	22.18	111.42	5.28	103.29
126	Pomiferin trimethyl ether	16.94	118.01	-5.50	108.85	1.06	112.86
127	Beta-LAPACHONE	23.88	119.48	20.71	103.83	8.34	122.06
128	Ursolic acid	7.39	117.64	10.71	112.21	6.37	120.14
129	Gossypol	-1.38	118.37	21.73	103.49	10.59	120.21
130	Pomiferin triacetate	-8.20	115.22	0.21	95.00	68.68	84.89
131	Tetrandrine	20.39	113.87	5.07	104.77	18.93	118.20
132	Helenine	31.62	94.16	1.82	102.03	30.21	103.00
133	Cedrelone	58.02	100.93	46.98	108.65	15.93	95.87
134	Dihydrocelastrol	97.74	114.53	100.00	92.56	100.00	75.29

135	Spiculisporic acid	2.73	119.92	18.72	109.59	-2.73	108.36
136	Dihydrogambogic acid	4.72	126.13	4.16	109.85	-7.35	82.84
137	Alpha-MANGOSTIN	11.22	128.27	4.18	110.42	12.87	120.88
138	Isocotoin	19.10	123.34	25.32	108.64	13.57	98.57
139	Totarol	1.01	121.36	-17.81	110.67	13.98	117.54
140	Methyl gambogate methyl ether	95.99	106.87	99.64	84.58	97.84	80.31
141	Mundulone	20.01	118.39	-9.20	117.52	18.92	109.63
142	Dimethyl gambogInate	30.77	110.85	31.39	107.28	11.30	110.07
143	Dehydrorotenone	7.13	102.54	3.51	98.22	15.11	111.54
144	Beta-AMYRIN ACETATE	1.95	113.02	16.13	109.17	4.86	106.99
145	2-meo-Me-enedio-BENZALDEHYDE	5.38	116.52	15.86	111.34	21.55	127.32
146	Tropine	5.83	119.55	15.86	108.16	-0.84	109.76
147	Cortexolone	-3.60	112.96	2.11	111.20	6.46	113.51
148	Phenacylamine hydrochloride	3.47	108.75	10.95	111.25	12.67	113.42
149	Rotenone	56.71	101.31	15.57	91.19	34.84	68.47
150	Celastrol	96.74	104.41	100.00	91.05	100.00	59.46
151	Osajin	-10.26	115.61	2.98	108.61	3.49	107.33
152	Pomiferin	7.41	113.24	-14.05	105.78	48.84	110.05
153	Dihydrocelastryl diacetate	97.00	100.70	97.40	93.04	96.99	86.0
154	Deguelin(-)	39.90	102.31	-7.90	107.31	28.68	106.86
155	Captan	39.92	91.71	-2.54	103.94	-6.78	84.84
156	Anthracene-1,4-dione	49.03	99.45	5.21	96.81	-11.69	99.25
157	1,4-naphthoquinone	54.79	94.95	49.79	103.64	11.95	92.84
158	Rosolic acid	19.07	102.60	12.28	104.87	10.02	87.95
159	Cadmium acetate	76.09	102.37	99.83	92.07	98.53	94.88
160	Calcein	-125.76	104.36	17.77	105.34	-27.37	98.31
161	Estriol benzyl ether	35.54	106.08	-17.76	107.28	15.77	112.51
162	1-benzylconhphetclmeco	11.56	97.12	0.37	95.59	-8.37	95.34
163	Berbamine hydrochloride	81.00	109.40	-36.86	99.12	23.82	101.22
164	Dronedarone hydrochloride	26.94	104.88	-15.01	99.05	74.92	107.83

Compounds showing >80% virus inhibition and >80% cell viability are indicated with coloured boxes. The 28 compounds that were selected for the tertiary screen are shown in red.

Table S2: Anti-CHIKV activity of the 28 compounds (tertiary screening).

Compound Name	CHIKV inhibition at 0.5 µM			CHIKV inhibition at 0.1 µM		
	ERMS	Vero	BHK-21	ERMS	Vero	BHK-21
Sanguinarium chloride	-	-	-	-	-	-
Pyrithione zinc	-	-	-	-	-	-
Disulfiram	+	-	-	+	-	-
Emetine dihydrochloride	+	+	+	+	-	+
Gentian violet	+	-	-	-	-	-
Gramicidin A	+	-	-	+	-	-
Thimerosal	+	-	-	-	-	-
Phenylmercuric acetate	+	-	-	-	-	-
Puromycin dihydrochloride	-	-	-	-	-	-
Suloctidil	-	-	-	-	-	-
Niclosamide	+	+	+	-	-	-
Monensin sodium	+	-	-	-	-	-
Cycloheximide	+	-	-	-	-	-
Mitoxantrone hydrochloride	-	-	-	-	-	-
Thiram	+	-	-	-	-	-
Penfluridol	-	-	-	-	-	-
Fluorescein	-	-	-	-	-	-
Chlorothalonil	+	+	-	-	-	-
Salinomycin	+	-	-	-	-	-
Patulin	-	+	-	-	-	-
Anthothecol	-	-	-	-	-	-
Gambogic acid	+	+	+	-	-	-
Dihydrocelastrol	+	-	+	-	-	-
Methyl gambogate methyl ether	+	-	+	-	-	-
Celastrol	+	+	+	-	-	-
Dihydrocelastryl diacetate	+	-	-	-	-	-
Cadmium acetate	-	+	+	-	-	-
Berbamine hydrochloride	-	-	-	-	-	-
**Total number of compounds showing anti-CHIKV activity (>80% virus inhibition)**	**17**	**7**	**7**	**3**	**0**	**1**

(green)	>80% virus inhibition in 3 different cell lines
(yellow)	>80% virus inhibition in 2 different cell lines

The four compounds that were selected for further studies are shown in red.

Table S3: SiteMap analysis.

Site	SiteScore	Size	Dscore	Volume	Exposure	Enclosure	don/acc	Residues
Site-1	1.019	187	1.056	708.981	0.71	0.689	1.217	S88, M91, V92, E95, V99, Q291, D155, L156, P159, P160, Y161, H162, F164, A165, S208, G209, K210, H162, T233, V234, D235, C284, L239, F255, A256, C257, H258, T261, K211, E212, P281, K282, Q283, G285, F286, F287, N288, M289, M292, V294, N297, H258, A380, A381, E408, H409
Site-2	0.88	63	0.837	148.862	0.58	0.744	0.417	V48, T52, A57, R9, R139, R140, A64, Y65, G67, R68, H19, M290, H19, M292, Y93, N94, R101, R96, E97, F98, N100
Site-3	0.831	47	0.854	131.369	0.612	0.651	0.621	H306, K307, S308, F185, G186, V187, H325, Y326, E327, K329, D280, P281, K282, H306, H325
Site-4	0.773	48	0.652	162.925	0.678	0.695	0.673	G384, T386, R311, R312, G189, S190, G191, K192, S193, A194, K197, AL 2003, F2004, F2005, E418, N213, E216, D220, Q224
Site-5	0.683	33	0.67	98.098	0.711	0.586	1.449	L435, P438, P439, K440, G441, N442, F443, K444, L315, P316, A319, N337, P339, I340

The table summarises the ED binding site characteristics for the CHIKV nsP2 identified using Schrödinger's SiteMap module. The SiteScore (score assessing the ligand-binding potential), Size (number of site points defining the pocket), Dscore (druggability score, complementing SiteScore), Volume (binding site volume in cubic angstroms), Exposure (solvent exposure, lower values indicate buried sites), Enclosure (degree of site enclosure, higher values suggest well-defined pockets), Don/Acc (hydrogen-bond donor/acceptor potential), and Residues (amino acids lining the site, critical for ligand interactions) for different sites are presented.
